# Genome-Wide Analysis of Serine Carboxypeptidase-Like Acyltransferase Gene Family for Evolution and Characterization of Enzymes Involved in the Biosynthesis of Galloylated Catechins in the Tea Plant (*Camellia sinensis*)

**DOI:** 10.3389/fpls.2020.00848

**Published:** 2020-06-25

**Authors:** Muhammad Zulfiqar Ahmad, Penghui Li, Guangbiao She, Enhua Xia, Vagner A. Benedito, Xiao Chun Wan, Jian Zhao

**Affiliations:** ^1^State Key Laboratory of Tea Plant Biology and Utilization, Anhui Agricultural University, Hefei, China; ^2^Division of Plant & Soil Sciences, West Virginia University, Morgantown, WV, United States

**Keywords:** acyltransferase, catechins, enzyme activity, galloylation, health function, specialized metabolism

## Abstract

Tea (*Camellia sinensis* L.) leaves synthesize and concentrate a vast array of galloylated catechins (e.g., EGCG and ECG) and non-galloylated catechins (e.g., EGC, catechin, and epicatechin), together constituting 8%–24% of the dry leaf mass. Galloylated catechins account for a major portion of soluble catechins in tea leaves (up to 75%) and make a major contribution to the astringency and bitter taste of the green tea, and their pharmacological activity for human health. However, the catechin galloylation mechanism in tea plants is largely unknown at molecular levels. Previous studies indicated that glucosyltransferases and serine carboxypeptidase-like acyltransferases (SCPL) might be involved in the process. However, details about the roles of SCPLs in the biosynthesis of galloylated catechins remain to be elucidated. Here, we performed the genome-wide identification of SCPL genes in the tea plant genome. Several SCPLs were grouped into clade IA, which encompasses previously characterized SCPL-IA enzymes with an acylation function. Twenty-eight tea genes in this clade were differentially expressed in young leaves and vegetative buds. We characterized three SCPL-IA enzymes (CsSCPL11-IA, CsSCPL13-IA, CsSCPL14-IA) with galloylation activity toward epicatechins using recombinant enzymes. Not only the expression levels of these SCPLIA genes coincide with the accumulation of galloylated catechins in tea plants, but their recombinant enzymes also displayed β-glucogallin:catechin galloyl acyltransferase activity. These findings provide the first insights into the identities of genes encoding glucogallin:catechin galloyl acyltransferases with an active role in the biosynthesis of galloylated catechins in tea plants.

## Introduction

Many plant metabolites, particularly specialized compounds, are subject to various modifications, such as glycosylation, malonylation, methylation, acylation, and prenylation (Bowles et al., 2005; Kosma et al., 2012; Bontpart et al., 2015; Ahmad et al., 2017). These modifications further diversify plant specialized metabolites and generate modified bioactive molecules with additional physicochemical properties, thereby creating new biological properties that the original metabolites do not have (Wang et al., 2019). Plant genomes evolved to owning a large number of gene families encoding modifying enzymes in order to adapt against adverse environments and survive frequent attacks by pathogens and herbivores (Wilson et al., 2016). Among these modifications, acylation is the most common and important of the modifications on plant metabolites, including phenolics, lipid barriers, sugars, and polyamines. The various modes of acylations enable plants to gain many functions in metabolic and physiological processes, as well as defense responses against abiotic or biotic stresses (Bontpart et al., 2015; Wilson et al., 2016).

Plant phenolics are ubiquitous specialized metabolites that contain a phenolic ring with at least one hydroxyl group (Bontpart et al., 2015). They are involved in growth, development, and defense responses. As the most extensively studied specialized metabolites in plants, their biosynthetic pathways are well known and involve multiple modification types, such as acylation, which culminates with the production of a vast diversity of phenolic products. Moreover, due to the high availability of simple phenolics in most plants, they often are not only the acceptor molecules during acylation but are also modified as energy-rich donors during other types of metabolite modifications in plants (Bontpart et al., 2015; Wilson et al., 2016). Two major acyltransferase families that use phenolic compounds either as acceptor or donor molecules have been described and characterized, each of which uses a distinct type of acyl donor (Bontpart et al., 2015). BAHD acyltransferases, named after the first four enzymes biochemically characterized in the family (BEAT, AHCT, HCBT, and DAT), have been extensively studied in a wide range of plant specialized metabolism and are characterized by using acyl-CoA thioesters as donor molecules (Schilmiller et al., 2012; Bontpart et al., 2015; Wilson et al., 2016; Zhang et al., 2018). On the other hand, serine carboxypeptidase-like (SCPL) acyltransferases use 1-*O*-β-glucose esters as acyl donors to facilitate the transacylation reaction to a large variety of phenolics, acids, saponins, and other compounds were only discovered recently (Mugford and Milkowski, 2012).

SCPLs revealed sequence homology to serine carboxypeptidases (Steffens, 2000; Milkowski and Strack, 2004; Bontpart et al., 2015). SCPLs can produce a variety of phenolic compounds, such as β-1-cinnamoyl-D-glucose, sinapoyl esters, and gallotannins (Wilson et al., 2016). However, not a single gene coding for an SCPL was characterized until the early 2000s (Lehfeldt et al., 2000; Li and Steffens, 2000). SCPLs provide biological relevance to various compounds, such as sinapoylglucose:malate sinapoyltransferase (Lehfeldt et al., 2000), sinapoylglucose:choline sinapoyltransferase (Shirley and Chapple, 2003), sinapoylglucose:sinapoylglucose sinapoyltransferase (Fraser et al., 2007), sinapoylglucose:antho cyanin sinapoyltransferase (Fraser et al., 2007) in Arabidopsis. These enzymes use 1-*O*-sinapoyl-β-D-glucose to form sinapoylmalate, sinapoylcholine (sinapine), 1,2-di-*O*-sinapoyl-β-D-glucose, and sinapoylated anthocyanins, respectively. Subsequently, two *Brassica napus* orthologs BnSCT1 and BnSCT2 were also characterized (Milkowski and Strack, 2004; Weier et al., 2008). Gallic acid is a trihydroxybenzoic acid that occurs in several dicot clades (Karas et al., 2017). Galloylated flavon-3-ols play vital roles in protecting plant cell membranes from oxidative damage (Saffari and Sadrzadeh, 2004). UDP-glucose:sinapic acid glucosyltransferase synthesizes sinapoylglucose in the Brassicaceae (Milkowski and Strack, 2004) whereas three UGTs form hydroxycinnamoyl glucose esters and β-glucogallin in grapevine (Khater et al., 2012), which are used by several SCPLs. Three grapevine (*Vitis vinifera*) glucosyltransferase genes *VvgGT1* to *3* were isolated and characterized (Khater et al., 2012). A purified polypeptide from tea leaves displaying epicatechin:1-*O*-galloyl-β-D-glucose *O*-galloyltransferase activity was previously reported (Liu et al., 2012). Galloyl-glucose esters, such as galloyl 1-*O*-β-D-glucose(β-glucogallin), is required to act as an acyl donor for the galloylation of flavon-3-ols. β-Glucogallin was produced from gallic acid by a UDP-glucosyltransferase in a crude protein extract of tea plant (*Camellia sinensis*) leaves (Liu et al., 2012). However, the gene encoding this enzyme is yet to be reported.

Catechins is the collective term for tea soluble flavan-3-ols, including six major types, (−)-epicatechin (EC), (+)-catechin (C), (−)-epigallocatechin (EGC), (+)-gallocatechin (GC), (−)-epicatechin-3-gallate (ECG), and (−)-epigallocatechin-3-gallate (EGCG) (Wei et al., 2018; Zhao et al., 2020). Among them, galloylated (e.g., EGCG and ECG) and non-galloylated catechins (e.g., EGC, C, and EC) together constitute 8%–24% of the dry leaf mass. As characteristic to teas, galloylated catechins account for up to 75% of the total catechins and make major contributions to the astringency and bitterness tastes of the green tea and numerous pharmacological activities for promoting human health (Zhao et al., 2020). Despite of their importance, the molecular and genetic bases of catechin galloylation remain largely unexplored. In tea plant leaves, several flavonoid 3′-hydroxylases and flavonoid 3′,5′-hydroxylases are two types of enzymes controlling the generation of EC and EGC, respectively, two major non-galloylated catechins (Wei et al., 2015). As precursors of galloylated flavan-3-ols and flavonols, β-glucogallin or polygalloylated glucoses are ubiquitous in the core eudicots (Moore et al., 2005; Liu et al., 2012; Wilson et al., 2016; Ciarkowska et al., 2018). Several enzymatic activities using β-glucogallin to synthesize gallotannins were observed in oak (*Quercus* spp.) (Niemetz and Gross, 2005). Moreover, β-glucogallin serves as a precursor for the biosynthesis of galloylated catechins, and it could be acylated with galloyl moieties in other species, such as tea plant, grapevine, and persimmon (*Diospyros kaki*). β-Glucogallin is formed from glucose and gallic acid in tea plants by the UDP-glucose:galloyl-1-*O*-β-D-glucosyltransferase CsUGT84A22 (Liu et al., 2012; Cui et al., 2016), which homologs were also identified in pomegranate (*Punica granatum*) and *Eucalyptus camaldulensis* (Ono et al., 2016; Tahara et al., 2018). β-Glucogallin is also used for the biosynthesis of proanthocyanidins via SCPLs VvGAT1 and VvGAT2 in grapevine (Terrier et al., 2009; Carrier et al., 2013; Koyama et al., 2014). Differential expression of the DkSCPL1 and DkSCPL2 genes in persimmon was observed in astringent versus non-astringent fruits (Ikegami et al., 2007; Akagi et al., 2009). In tea leaves, galloylated catechins not only account for most soluble catechins, but are also the prominent contributors to flavor, such as astringency and bitterness, and have higher pharmacological activity for human health than non-galloylated catechins (Ikegami et al., 2007; Akagi et al., 2009; Hayashi et al., 2010; Wilson et al., 2016). β-Glucogallin (β-G) serves not only as an acyl donor in successive transacylation steps to yield hydrolysable tannins (HTs), such as substituted di- through penta-galloylglucose derivatives (Niemetz and Gross, 2005). These HTs may also be used as acyl donors for the generation of galloylated catechins (Bontpart et al., 2015; Wilson et al., 2016; Ciarkowska et al., 2018). Assays with β-G:1,2,3,6-tetra-*O*-galloyl- and β-D-glucose-4-*O*-galloyltransferase activities were also performed in pedunculate oak (*Quercus robur*), although their coding gene is not cloned (Grundhöfer and Gross, 2001).

Similar to HTs, galloylated catechins can be hydrolyzed to non-galloylated catechins and gallic acid by a galloylated catechins hydrolase, which has been widely characterized in bacteria at the catalytic as well as genetic levels (Niemetz and Gross, 2005; de las Rivas et al., 2019). Interestingly, no 1,2,3,4,6-pentagalloylglucose was identified in tea plants, suggesting that the hydrolyase activity toward this compound is very active. More than 30 gallic acid derivatives, including two types of HTs (gallotannins and ellagitannins), as well as several types of polygalloylated glucose (PGG), such as 1,6-digalloylglucose, 1,2,6-trigalloylglucose, 1-galloylglucose, 1,2-digalloylglucose, 6-galloylglucose, 2,6-digalloylglucose, 1,3-digalloylglucose, 1,2,3,4-tetragalloylglucose, and 1,4,6-trigalloylglucose have been identified in tea plant leaves (Yang and Tomás-Barberán, 2018; Wei et al., 2019).

Here, we explored the tea genome and identified novel putative SCPL genes involved in the generation of galloylated catechins in the leaves. We further characterized three SCPL enzymes, which produced *in vitro* galloylated catechins with PGG as an acyl donor and catechins as acceptor substrates. This study provides new insights into the understanding of this important gene family involved in the modification of tea catechins, which are of high relevance in the food chemistry and pharmacological industries.

## Materials and Methods

### Identification of SCPL Family Genes in the *Camellia sinensis* Genome

BLASTP against the *Camellia sinensis* proteome dataset obtained from the genome sequence (Wei et al., 2018; The Tea Plant Information Archive – TPIA public database^1^) was used to search for SCPL-coding genes with characterized protein sequences as queries. The SCP motif (PF00450), was searched through Pfam in each retrieved sequence to confirm it as a putative SCPL protein. Features of SCPL proteins, such as the number of amino acid residues, gene architecture (exon/intron arrangement) and start-to-end position of their respective genes in the genome, were retrieved from the TPIA annotation. Physical parameters of each gene product, such as molecular weight (Mw) and isoelectric point (pI), were calculated using the ExPASy tool Compute pI/Mw^2^ using default parameters.

### Chromosomal Position, Phylogeny, and Gene Structure Analysis of SCPL Genes

PhenoGram Plot^3^ used to create the image of scaffold locations of SCPL genes using the information available at the TPIA database. A phylogenetic analysis with functionally characterized proteins from different species and all SCPL proteins encoded in the *C. sinensis* genome was conducted to explore the evolutionary relationships of this gene family. An unrooted phylogenetic tree was constructed following the Neighbor-Joining method involving 1,000 replicates with the bootstrap test in MEGA 6.0 (Tamura et al., 2013). The Gene Structure Display Server v.2.0 tool (Hu et al., 2015^4^) was used to determine the exon/intron structures of the tea SCPL genes.

### Calculation of K_a_ and K_s_ Ratios and Evolutionary Selection Analysis

Synonymous and non-synonymous substitution rates (K_s_ and K_a_, respectively) of two closely related CsSCPL genes, along with their K_a_/K_s_ ratios, were calculated online^5^ to evaluate the selection mode of each CsSCPL paralogous gene pair identified. Assumptions of negative, neutral, and positive evolution were considered for ratios < 1, = 1, and > 1, respectively. The evolutionary signals of positive sites were determined with FEL, IFEL, REL and SLAC tests through DATAMONKEY^6^. For the evolutionary selection analysis, the phylogeny was reconstructed through the HyPhy program with CsSCPL cDNA sequences as input and the Nei-Gojobori method in MEGA 6. Maximum-likelihood based on the Kimura-2 parameter model was used to infer the evolutionary gene histories. The detection of episodic diversification of individual coding sites was performed through different approaches. Episodic diversifying selection and pervasive positive selection were identified at the individual branch site level using the Mixed-Effect Model of Evolution (MEME). The Markov Chain Monte Carlo (MCMC) method was used in the Fast, Unconstrained Bayesian Approximation analysis to ensure the strength over predefined sites via the approximate Bayesian method (Murrell et al., 2012). The parameter ω = β/α was used in MEME to fit the data in the GTR nucleotide model as initial values. The parameters β:β^–^ ≤ α and β^+^ were used to measure the selection pressure, whereas β^–^, β^+^ and α were used to estimate the variability of site-to-site substitution rate. The Likelihood Ratio Test (LRT) based on χ^2^ asymptotic distribution was considered significant at *p*-value < 0.05.

### Identification of Conserved Motifs and Promoter Region Analysis

The analysis of conserved regions within CsSCPL family genes was executed through MEME^7^, and their genomic assemblies were screened through the Pfam database^8^. Regulatory elements in the 1.5-Kb promoter region upstream of the start codon for each CsSCPL gene were identified using the Plant *cis*-acting Regulatory DNA Elements (PlantCARE) program^9^.

### *In silico* Gene Expression Analysis

Public gene expression data of identified CsSCPL genes in seven tissues (apical vegetative bud; young, middle and old leaves; root; stem; and fruit: Supplementary Dataset S3) were retrieved from the microarray-based transcriptome tea data for the cultivar Shuchazao (Wei et al., 2018).

### Plant Growth Conditions and Treatment Experiments

Tea (*Camellia sinensis* var. *sinensis* cv. Shuchazao) seeds germinated in vermiculite in a growth chamber at 22 ± 2°C. For the aluminum (Al^3+^) treatment, one-year cuttings were hydroponically grown in Shigeki Konishi solution (Supplementary Table S1) under acidic conditions (pH 5.0, 0.4 mM Al^3+^: control) or Al^3+^ exposure (pH 4.0, 2.5 mM Al^3+^) in a growth chamber set with light period 16 h/8 h (light/dark) and temperature at 23°C. Root systems were collected at 0 h, 12 h, 24 h, and 48 h (Wang et al., 2017) for gene expression analysis. MeJA treatment experiments were followed as described previously (Shi et al., 2015): detached branches with tender tea shoots were sprayed with 100 μM Methyl Jasmonate (MeJA) solution, while the controls were treated with distilled water. Tea leaves were respectively collected at 0 h, 12 h, 24 h, and 48 h. The polyethylene glycol (PEG) and NaCl exposure experiments were performed as previously (Zhang et al., 2017): 25% PEG or 200 mM NaCl were used respectively to simulate plant drought- and salt-stress conditions for 0 h, 24 h, 48 h, and 72 h. For the cold treatments, tea plant leaves were collected during the cold accumulation (CA) process. Control: 25°C; CA1.6 h: 10°C for 6 h; CA1.7days: from 10°C to 4°C for 6 days; CA2.7days: from 4°C to 0°C for 7 days; DA.7d: recover under 25°C to 20°C for 7 days. For the shading treatment, tea plants cultivated in the 20-year-old tea garden in Anhui, where plants grow under natural condition (full sun exposure) or 70%–90% shading provided by a net cover. Buds were collected at 0 h, 4 h, 8 h, 2 days, 4 days, 8 days, and 14 days as previously reported (Liu et al., 2018).

### RNA-Seq Data Analysis, and Gene Expression Analysis, and Validation With qRT-PCR

Tea plant tissues were sampled, rinsed immediately, snap-frozen in liquid N_2_, and stored at −80°C for RNA extraction. Total RNA was performed with RNA extraction kits (BIOTECH, Beijing, China). The libraries were synthesized using Illumina HiSeq2500 library prep kits according to the manufacture’s protocol. RNA-Seq (PE150bp) sequencing was performed on an Illumina HiSeq2500 platform. A total clean dataset of about 6 Gb (Q30 ≥ 80%) was obtained for each biological sample for analysis according to the tea plant genome sequence and annotation (Wei et al., 2018). Reads Per Kilobase of transcript per Million mapped reads (RPKM) and read counts were calculated using eXpress. The analysis of differential gene expression followed the method previously published (Ahmad et al., 2020).

Transcriptome data for the cv. Shuchazao were retrieved from the tea information archive^10^. Fragments per kilobase of exon per million fragments (FPKM) values were used to estimate gene expression in eight tea plant tissues (root, stem, old leaf, mature leaf, young leaf, apical bud, flower and fruit) and various treatments. The expression levels of *CsSCPL1A-AT* genes were calculated using Log_10_(FPKM). The data were utilized to quantify the expression of *CsSCPL* genes in the roots of hydroponically growing tea cuttage seedlings in response to Al^3+^ and MeJA stresses (Supplementary Dataset S3). Mev4.9.0^11^ was used to display the expression data in heatmaps. Expression and metabolite association analyses for *CsSCPL1A* genes potentially associated with galloylated catechins biosynthesis were carried out as in previous studies (Shi et al., 2015; Liu et al., 2018; Wei et al., 2018).

Pearson correlation analysis on *CsSCPL-I* gene expression and the contents of catechins from multiple tissue experimental datasets. R package also was used to evaluate the correlation between gene expression and metabolism and global gene expression. The resulting heatmap was structured by pHeatmap R package. Dark red means positive correlation values, and light red means negative correlation. Transcriptome and metabolic profiling data sets were from different tissues of tea plants (Wei et al., 2018).

Based on their expression dynamics, five tea CsSCPL-I genes [i.e., *CsSCPL2-I* (TEA009664, NCBI Genbank accession MK843824), *CsSCPL5-I* (TEA034028; MK843825), *CsSCPL11-I* (TEA023451; MK843826), *CsSCPL13-I* (TEA034055; MK843827), and *CsSCPL14-I* (TEA027270; MK843828)] were selected for validation of their stress responses in different tissues via qRT-PCR. The gene-specific primers used listed in Supplementary Dataset S4. An iQ5 Real-Time PCR System (Bio-Rad) was used with 96-well plates and 20-μL reaction volume. Each reaction consisted of 2.5 μL 2X Power SYBR Master Mix (Applied Biosystems), 1 μL primer mix (0.4 μL of 10 mM each primer + 0.2 μL H_2_O), and 2 μL cDNA reaction diluted 1:30. The gene expression was normalized using the housekeeping *CsACTIN* (TEA002341) gene.

### Cloning and Expression of CsSCPL Recombinant Proteins and Enzyme Activity Assay

The open reading frames (ORF) of the five CsSCPL-I genes listed above were amplified with gene-specific primers. Amplification was performed on a Mastercycler PCR equipment (Eppendorf) using 0.5 μL ExTaq DNA polymerase (Takara) and 2.0 μL cDNA dilutions (1:30) as templates. The gel-purified PCR products inserted into the pGEM-T easy vector (Promega), cloned into *E. coli*, and confirmed by sequencing. For expression of functional SCPL proteins, the truncated CsSCPLs, encoding by the ORFs of CsSCPL11-IA and CsSCPL14-IA with a deletion of the first 150 bp and an ATG codon insertion and full length CsSCPL13-IA (Supplementary Figure S2B), were cloned into pDONR221 (Invitrogen) followed by recombination into pDEST17 with LR Recombinase (Invitrogen). The constructs transferred into *E. coli* strain Rosetta for heterologous protein induction. The single colony of confirmed construct taken and overnight grown in 10 mL LB media with ampicillin (50 μg mL^–1^) at 37°C. Bacterial cultures grown overnight were diluted in 300 mL LB media (1:100) with ampicillin (50 μg mL^–1^) and kept in a shaker at 37°C. When the OD_600_ reached 0.8 to 1.0, 0.2 mM isopropyl 1-β-D thiogalactoside (IPTG) was added to the culture and kept at 25°C. After a 10-h incubation period, the cultures were collected, centrifuged at 12,000 rpm for 30 min and re-suspended in 20 mL lysis buffer [200 mM Tris–HCl (pH 8.0), 0.1% Triton X-100, and 5 mM β-mercaptoethanol] and kept on ice for 1h. The cells were then broken through ultrasonication and centrifuged at 12,000 rpm for 30 min at 4°C. Supernatants were collected and purified using a nickel-resin purification kit (Promega). Protein extracts were examined through sodium dodecyl sulfate-polyacrylamide gel electrophoresis (SDS–PAGE).

For the enzyme activity assays, 50-μl total reaction mixtures containing 50 mM potassium phosphate buffer (pH 6.0), 1.0 mM catechin (C), epicatechin (EC), gallocatechin (GC), epigallocatechin (EGC), 0.4 mM 1,2,3,4,6-penta-*O*-galloyl-β-D-glucose (PGG), and 1.0 μg purified recombinant enzyme were used to verify the gallate activity of each selected CsSCPL. The reactions were carried out at 30°C for 20 mins and stopped by adding an equal amount of 100% methanol. The samples were analyzed by high-performance liquid chromatography (HPLC) and liquid chromatography-mass spectrometry (LC-MS), as described previously (Wei et al., 2018).

### Analyses of Enzyme Kinetics

The *K*_M_ and *V*_Max_ kinetic parameters were obtained through Lineweaver-Burk plots for *CsSCPL11-IA*, *CsSCPL13-IA*, and *CsSCPL14-IA* with various concentrations of EC and EGC as acceptors (0.5 to 50 mM) and PGG (0.4 mM) as the donor substrate, in a total reaction volume of 50 μL. The PGG kinetic parameters for these recombinant proteins were also measured with the PGG (0.1 to 10.0 mM) as the acyl donor and EGC (fixed concentration at 1.0 mM) as the acceptor substrate. The reactions were analyzed on HPLC.

### Statistical Analyses

At least 3 biological replicates were used in this study with at least 2 repetitions to obtain the data for analysis. Differences between paired data from the enzymes and controls under various conditions were analyzed via ANOVA followed by the Student’s *t*-test (*n* = 3).

## Results

### SCPL Gene Mining in the *Camellia sinensis* Genome

A total of 47 SCPL-related genes were identified in the *C. sinensis* genome and annotated according to their distribution in different phylogenetic classes (Supplementary Dataset S1). Their coding regions ranged from 717 bp (TEA034030) to 1,758 bp (TEA034034) with an average of 1,369 bp. The molecular weight of the CsSCPL gene products ranged between 27.49 kD (TEA034030) and 73.8 kD (TEA034056) and averaged 51.41 kD. The CsSCPL pI values ranged from 4.67 (TEA010715 and TEA016469) to 9.97 (TEA034027), with 77% (36/47) having acidic pI values. The subcellular localization of a protein influences its function by controlling the ability to obtain and use all types of molecular interacting partners. So, protein localization is an important piece of information in creating hypotheses about cellular functions of newly discovered proteins (Scott et al., 2005). About 61% of the CsSCPL-I proteins are predicted to localize to the plasma membrane, whereas 65% of the CsSCPL-II proteins are estimated to localize to the lysosome. Meanwhile, the remaining members of CsSCPL-I and CsSCPL-II clades are estimated to be found in other organelles, such as the lysosome, nucleus and cytoplasm, as well as extracellularly (Supplementary Dataset S1). Details on the products of each CsSCPL gene have shown in Supplementary Dataset S1.

### Phylogeny and Motif Analysis of CsSCPLs

**FIGURE 1:**
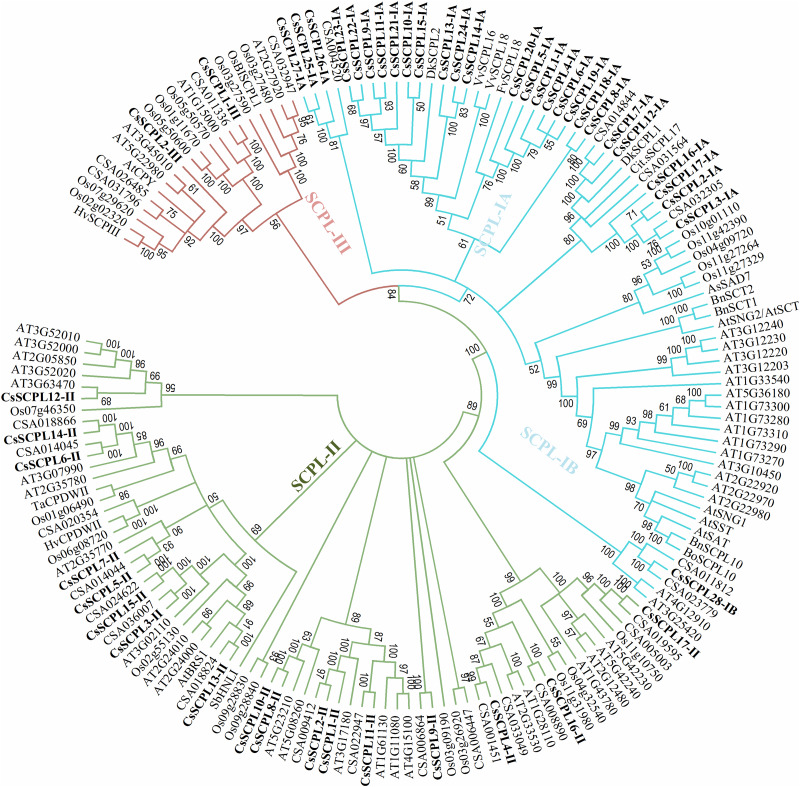
Phylogenetic tree of tea SCPL family genes with characterized SCPL genes from different plant species. The tree was constructed with MEGA 6.0 using the Neighbor-Joining (NJ) method. Bootstrap values in percentage (1,000 replicates) are indicated on the nodes. The subclades are identified with different colors: SCPL-I (blue), SCPL-II (green), and SCPL-III (maroon).

**FIGURE 2:**
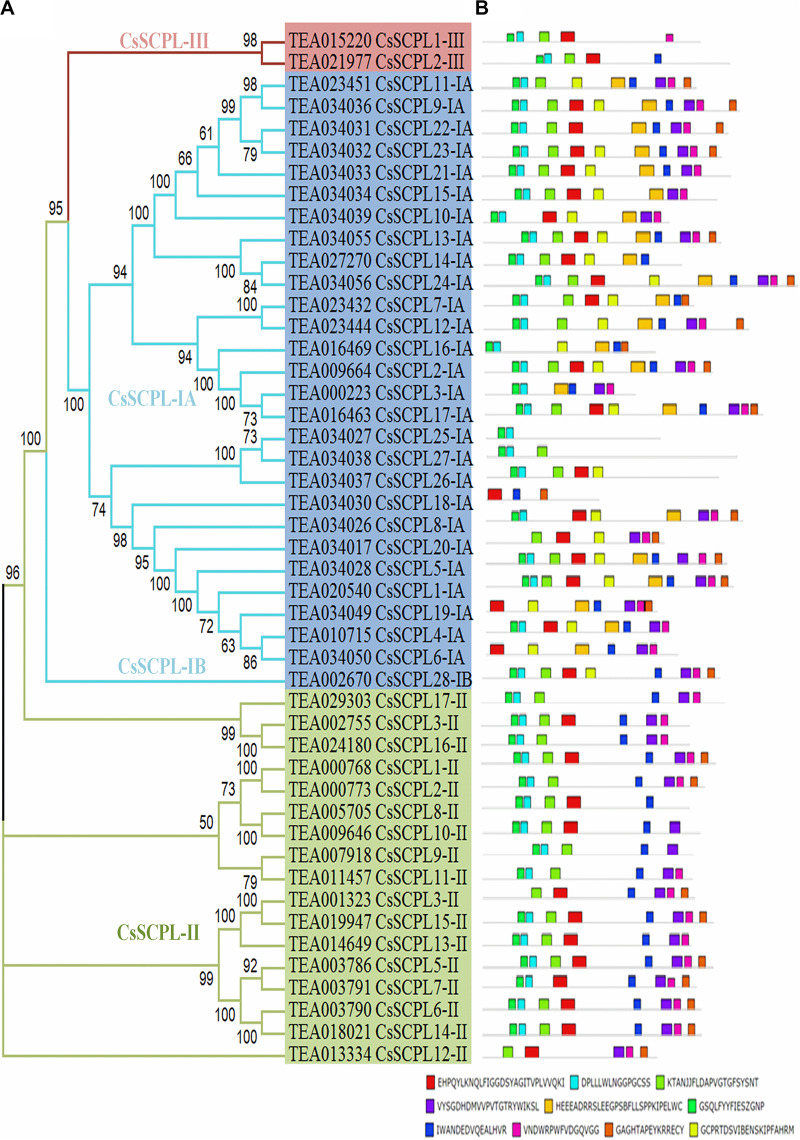
Phylogenetic analyses and conserved motif analysis of tea CsSCPL genes. **(A)** The phylogenetic tree of CsSCPLs genes was constructed through MEGA 6.0 using the Neighbor-Joining (NJ) method. Bootstrap values in percentage (1,000 replicates) are indicated on the nodes. The subclades are identified with different colors: SCPL-I (blue), SCPL-II (green), and SCPL-III (maroon). **(B)** The identified motifs of ScSCPL proteins are shown as colored boxes. The CsSCPL proteins are listed according to their phylogenetic relationships.

A phylogenetic tree was constructed from tea protein sequences as well as functionally characterized SCPL sequences from other plant species and the SCPLs from rice and Arabidopsis genomes in order to evaluate the evolutionary relationships among them (Figure 1). According to the classification and structural features of SCPL proteins from previous studies in rice and Arabidopsis models, CsSCPL genes are divided into three classes: CsSCPL-I to CsSCPL-III. CsSCPL-I is further split into two subclasses: CsSCPL-IA and CsSCPL-IB (Figure 1). The annotation of all 47 tea SCPLs was carried out according to their phylogenetic position: 27 fell into the CsSCPL-IA clade (CsSCPL1-IA to 27-IA) whereas only one member fell into CsSCPL-IB (CsSCPL28-IB). The CsSCPL-II clade contains 17 tea proteins (CsSCPL1-II to 17-II), and CsSCPL-III is limited to only two members (CsSCPL1-III and 2-III) (Figure 2A). Tea CsSCPL-I members intermixed with many SCPLs from other species but diverged from most Arabidopsis SCPLs. The conserved domains of CsSCPL proteins have determined by MEME (Figure 2B). In total, ten motifs have found, and their annotations confirmed through the Pfam and SMART databases (Letunic et al., 2012). The first four motifs are specific to serine carboxypeptidase enzymes and found in virtually all CsSCPLs (Supplementary Table S2; Figure 2B). All CsSCPLs motifs were generally well conserved but especially similar within the same phylogenetic class. In addition, each clade contained unique motifs – for example, motifs 5 and 10 were only found in the CsSCPL-I clade, which suggests that they have distinct features and potential specific functions related to each class.

### Exon/Intron Organization Within CsSCPL-I Genes

**FIGURE 3:**
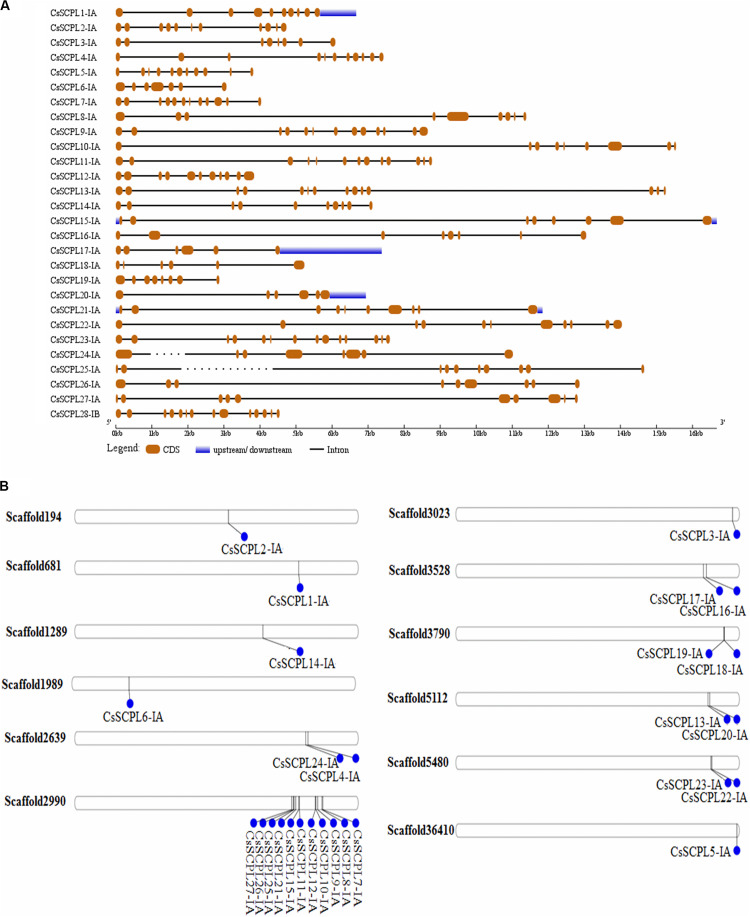
Gene architecture of tea CsSCPL genes. **(A)** The gene structures of CsSCPLI were plotted using red boxes representing exons, black lines representing introns, and blue boxes indicating UTR sequences. The scale in the bottom is in the unit of kilobase (kb). **(B)** Scaffold locations and duplications of CsSCPL-I genes. Scaffold numbers are indicated on leftside of each respective scaffold. The different genes located according to their presence in bps.

The exon/intron organization analysis of CsSCPL-I genes was performed to understand their structural diversity (Figure 3A). Extensive variation was found in exon numbers within the CsSCPL-I class, ranging from 6 (*CsSCPL18-IA* and *20-IA*) to 14 exons (*CsSCPL13-IA* and *23-IA*), with an average of 10 exons per gene (Supplementary Dataset S1; Figure 3A). About 11% (3/28) of the CsSCPL-I genes has 8 exons, 21% (6/28) contains 9 exons, 14% (4/28) has 10 exons, another 21% (6/28) shows 11 exons, while the remaining (40%, 11/28) contains 12 to 14 exons (Supplementary Dataset S1; Figure 3A). The comparison of gene architectures between each of the five CsSCPL paralogous pairs identified revealed that only one pair (CsSCPL27-IA/25-IA) contained the same number of exons.

### Chromosomal Distribution and Evolutionary Analysis of CsSCPL-I Coding Sequences

The 27 CsSCPL-IA genes were unevenly distributed on twelve different scaffolds of the tea genome assembly (Figure 3B). Interestingly, while most scaffolds contained one or two CsSCPL-I genes, scaffold2990 contained 11, with the majority clustering together in the phylogenetic tree (Figure 1). The expansion of gene families and their neo/sub-functionalization is a common phenomenon in plants and occurs through both, segmental as well as tandem gene duplication (Zhang and He, 2005; Kong et al., 2007). From the phylogenetic analysis, we conclude that the primary method for gene duplication of the CsSCPL-IA clade was segmental duplication while a single locus holds tandem duplications. We also found tandem duplications in two paralogous pairs (*SCPL9-IA/21-IA* and *12-IA/-IA*) along with segmental duplications in five pairs (*SCPL16-IA/20-IA, 13-IA/24-IA, 22-IA/10-IA, 6-IA/14-IA*, and *19-IA/1-IA*) (Figure 3B).

The selection history of coding sequences can be assessing through the K_a_/K_s_ ratio. In order to investigate the divergence of duplicated CsSCPL-IA members, K_a_ and K_s_ values, as well as the K_a_/K_s_ ratios, were determined for each CsSCPL-IA paralogous pair. K_a_/K_s_ ratios < 1, > 1, or = 1 indicate that the paralogous pair are respectively under purifying (negative), positive, or neutral selection (Juretic et al., 2005). The K_a_/K_s_ ratios of the seven CsSCPL-I paralogous pairs were between 0.88 and 1.67 (Supplementary Table S3), suggesting that distinct selection forces are acting on each pair. The K_a_/K_s_ ratios of tandemly duplicated gene pairs were 0.88 and 1.31 (Supplementary Table S3), indicating that these pairs are also undergoing different selection pressures. On the other hand, all segmentally duplicated paralogous pairs are under strong positive selection pressure (K_a_/K_s_ between 1.18 and 1.67), except for *SCPL6-IA/14-IA* and *19-IA/1-IA*, which are under purifying selection pressures (Ka/Ks 0.89 and 0.88, respectively) (Supplementary Table S3).

In order to maintain protein structure and function, conservation of amino acid positions during evolution is essential. Exploring this feature may clarify the selection pressures at work. The variation in substitution rates estimated by the MEC model adaptive selection test revealed that several SCPL protein regions are under positive selection (Supplementary Figure S1A). Over 5% (26 out of 476) CsSCPL-I amino acid residues are under positive selection, whereas the remaining are under purification (Supplementary Figure S1A). For CsSCPL-II proteins, all residues are under purifying selection (Supplementary Figure S1B).

For further corroboration of the selection pressures, we tested additional models. CsSCPL-I and CsSCPL-II members show different average ω ratios across their coding sites. The ω ratio output was CsSCPL-I > 1, and CsSCPL-II = 1 (Supplementary Table S4). This result suggests that most of the CsSCPL-I coding sites were positively selected and contain conserved amino acid residues exposed to purifying selection. Comparison of the M7 and M8 models was performed to refine the selection test. M8 fit the data more significantly than M7. Both classes, CsSCPL-I and CsSCPL-II, displayed positively selected sites in M8 for 7% of the sites under a ω value of 6.2 for CsSCPL-I, and a very minute proportion of 0.001% of CsSCPL-II sites was under a ω value of 1.0 (Supplementary Table S4). Therefore, the number of positively selected sites were significantly higher in the M8 model for CsSCPL-I than CsSCPL-II. Different likelihood-based approaches disclosed that 19 coding sites of CsSCPL-I genes but only 3 sites of CsSCPL-II genes were under positive selection (Supplementary Table S4). Various tests (FEL, IFEL, REL and SLAC) were used to identify evolutionary signs of positive selection through the computation of ω values. Each analysis detected a different number of coding sites under positive selection: for CsSCPL-I genes, FEL, IFEL, REL and SLAC revealed six, ten, fourteen, and seven coding sites respectively. Meanwhile, the respective numbers were seven, eleven, one, and four sites for CsSCPL-II genes (Supplementary Table S4). All of these tests were significant at a *P*-value < 0.05, except for REL, which detected sites under positive selection through a Bayes factor > 20. Therefore, reliable indication of positive selection of many CsSCPL-I gene sites and only a few of CsSCPL-II was consistently found throughout these analyses.

### Positive Selection Based on Amino Acid Positions

The identification of evolutionarily conserved amino acid positions has a vital role in understanding protein structure and function. The Bayes method was used to reveal functionally relevant sites through posterior probabilities. Amino acid positions with ω ≥ 1 indicate positive selection. The average length of 1,377 amino acid residues of CsSCPL-I was analyzed in BEB, which revealed 19 sites under positive selection. Meanwhile, only three positions were under positive selection for the CsSCPL-II average length of 1,370 amino acid residues (Supplementary Table S5). This result suggests that the identification of coding sites may explain the selection pressures and points out to functionally relevant amino acid residues in the SCPL proteins.

### *Cis*-Element Analysis of *CsSCPL-I* Gene Promoter Sequences

**FIGURE 4:**
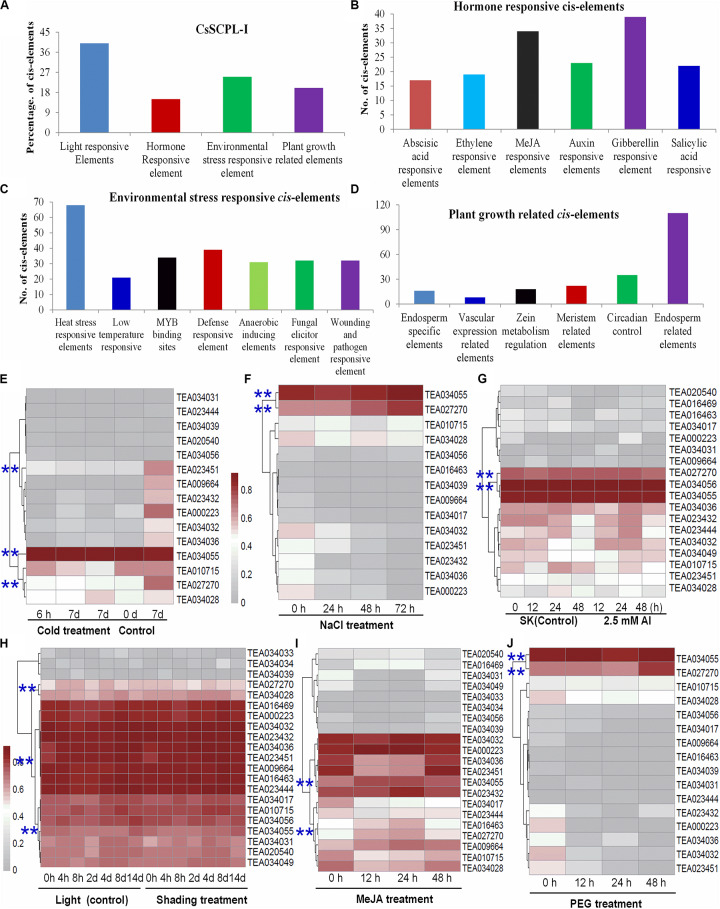
Environmental and hormonal regulation of CsSCPL genes in tea plants. PlantCare were used to analyze the 1,500 bp upstream region of each *CsSCPLI* gene. **(A)** The percentage of light responsive elements, hormone responsive elements, environmental stress related elements, and plant growth responsive elements in all *CsSCPLI* family members. **(B)** Different hormone (ABA, ethylene, MeJA, auxin, gibberellinlic acid) responsive elements in *CsSCPLI* genes *cis*-element regions. **(C)** Different environmental stress (heat, cold and dehydration, drought, defense, anaerobic, wound and pathogen) related elements in *CsSCPLI* genes *cis*-element regions. **(D)** Different plant growth related elements in *CsSCPLI* genes *cis*-element regions. **(E–J)** Heatmaps displaying expression patterns of various CsSCPL1A-AT genes under various conditions. Transcriptome data from experiments with tea cv. Shuchazao were retrieved from the tea plant information archive (http://tpia.teaplant.org/index.html). The expression levels of CsSCPL1A genes were normalized as fragments per-kilobase of exon per million fragments (FPKM) in eight tea plant tissues (root; stem; old, mature, and young leaves; apical bud; flower; and fruit) and displayed as Log_10_(FPKM) in heatmaps using Mev4.9.0 (https://sourceforge.net/projects/mev-tm4/).

The exact functions played by CsSCPL-I proteins in plants remain ambiguous. Characterizing the expression of a given gene in time and space is particularly useful to define its function. The dynamics of gene transcription in each cell as responses to environmental stimuli as well as internal cues are ultimately controlled by a modular composition of *cis*-elements present in gene promoter regions. Promoter sequences of *CsSCPL-I* genes were extracted and submitted to PlantCARE for *cis*-regulatory element identification (Lescot et al., 2002). Numerous elements were found in the 1.5-Kb upstream region of CsSCPL-I genes (Figures 4A–D; Supplementary Dataset S2). Twenty-six out of the 28 *CsSCPL-I* genes contained promoters with elements responsive to light (about 40% of the genes), hormones (15%), environmental stresses (25%), and plant growth (20%) (Figure 4A). Moreover, we identified binding sites for transcription factors that regulate responses to several hormones (Figure 4B). Among the hormone-response elements, these sensitive to GA were the most abundant in *CsSCPL-I* promoters, followed by MeJA. In addition to light as the most abundant factors may affecting *CsSCPL-I* genes, other abiotic and biotic stresses also impact on their expression significantly (Figure 4C). The maximum number of *cis*-elements related to plant growth found in the analysis was associated with the endosperm (Figure 4D).

The distribution pattern of *cis*-elements differed among *CsSCPL-I* members. For example, *CsSCPL7-IA* and *12-IA* promoters contained most ERE-related elements (five), whereas 62% of the *CsSCPL-I* gene promoters contained none (Supplementary Dataset S2). Remarkably, the single motif responsive to ABA was found in the *CsSCPL24-IA* promoter. The greatest number of heat-shock elements (eight) was found in *CsSCPL9-IA* and *22-IA*, followed by six in *CsSCPL11-IA*. A total of six defense-related elements was found in the *CsSCPL13-IA* promoter region (Supplementary Dataset S2). *CsSCPL11-IA, 13-IA*, and *14-IA* were differentially expressed in leaves or roots under the various treatments (Supplementary Dataset S3). Shading leads to a significant decreased in several galloylated catechins, such as EGCG and GCG (Liu et al., 2018). *CsSCPL13-IA*, *24-IA*, and *14-IA* expression levels were noticeably repressed in shaded leaves compared to plant under full light (Supplementary Dataset S3). However, most of the other SCPL genes were unchanged or even induced, such as *SCPL11-IA* (Figure 4; Supplementary Dataset S3). This result indicates that *CsSCPL11, 13, 14*genes are most likely responsible for the decreased levels of galloylated catechins under shading (Liu et al., 2018). Moreover, both MeJA and NaCl treatments promoted EGCG accumulation in the leaves or roots (Shi et al., 2015; Zhang et al., 2017). Our experiments showed that both of these conditions substantially induced the expression of *CsSCPL13-IA* and *14-IA* (Figure 4; Supplementary Dataset S3). These genes were also activated by PEG and Al exposure in roots (Figure 4), which is consistent with increased galloylated catechins previously reported (Zhang et al., 2017).

### *CsSCPL* Gene Expression Profiling

**FIGURE 5:**
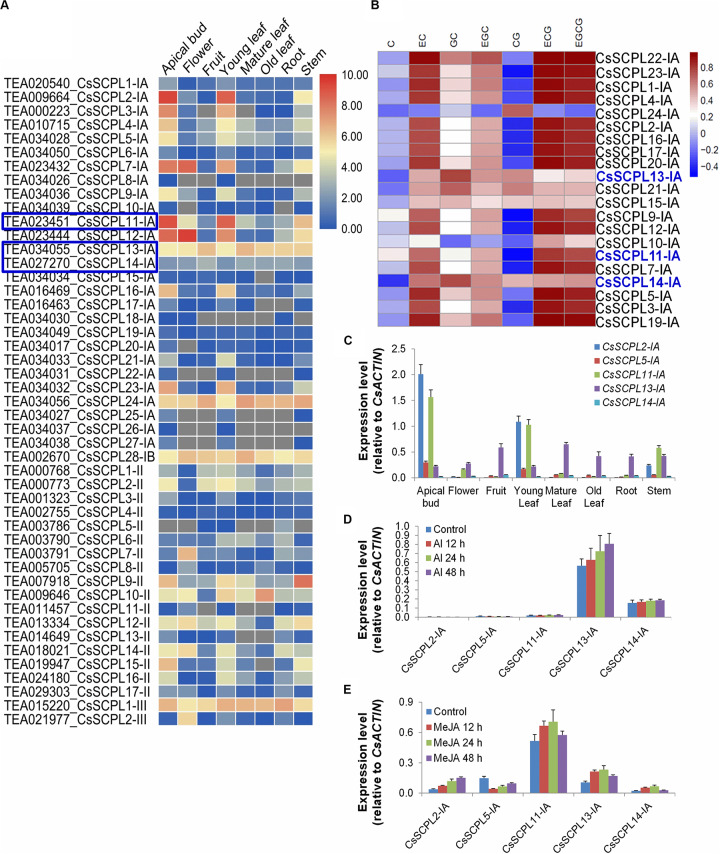
Expression patterns of CsSCPL genes in different Tea tissues and qRT-PCR confirmation. **(A)** The expression levels of CsSCPL genes in different tea tissues retrieved from a public database (Tea Plant Information Archive, http://teaplant.org/) and represented by constructing the heat map using TBTools program. **(B)** Correlation analysis of the contents of catechins of non-galloylated or gallolated in tea plant tissues with the expression levels of CsSCPL-IA genes. *CsSCPL-I* expression data and catechins contents from multiple tissue experiments (Wei et al., 2018). R package was used to evaluate the correlation. Color bar shows Pearson coefficiency values. **(C–E)** Expression of selected CsSCPL-I genes via RT-qPCR in different tea tissues **(C)**, and in response to Al^3+^ **(D)** and MeJA **(E)** exposures. Analytical data were obtained from atleast three biological replicates and are expressed as means ± SD.

We first examined the expression pattern of the 47 CsSCPL genes identified by analyzing publicly available RNA-Seq data from eight *C. sinensis* tissues (apical bud; flower; fruit; young, mature, and old leaves; root; and stem) (Figure 5A, Supplementary Dataset S3). Most genes showed expression in almost all tissues, with a few exceptions. About 25% (12/47) genes showed no or very low expression in all tissues. These genes may be induced by particular conditions or undergoing pseudofunctionalization. Additionally, *CsSCPL2-IA, 3-IA, 22-IA*, and *23-IA* were only expressed in the apical bud, young leaf, and stem. On the other hand, *CsSCPL5-IA, 11-IA, 13-IA, 14-IA*, and *24-IA* were highly expressed in all tissues analyzed (Figure 5A). The comparative expression analysis of paralog pairs showed conflicting results. Whereas three of the identified pairs showed very similar expression patterns, each member of the pairs Cs*SCPL13-IA/24-IA, 22-IA/10-IA*, and *19-IA/1-IA* showed distinct expression patterns (Figure 5A, Supplementary Dataset S3). This result is evidence that each paralogous member underwent or still are undergoing functional divergence.

Pearson correlation analysis between *CsSCPL-I* gene expression levels and the contents of catechins from multiple tissue experiments (Wei et al., 2018), showed that the experession of most *CsSCPL-I* genes was correlated with at least one of these catechins, indicating the redundant functions of *CsSCPL-I* genes on catechins biosyntesis (Figure 5B). Based on tissue-specific expression patterns and presence of promoter *cis*-elements responsive to different stresses, five CsSCPL-I genes were chosen for further analysis via qRT-PCR in seven tissues: *CsSCPL2-IA*, *5-IA*, *11-IA*, *13-IA*, and *14-IA* (Supplementary Dataset S3). *CsSCPL2-IA* and *11-IA* expressed relatively high in the apical bud and the young leaf (Figure 5C). *CsSCPL13-IA* expression was relatively constant across all tissues, whereas *CsSCPL14-IA* expressed very low in all tissues tested. Since our promoter analysis revealed several *cis*-elements associated with MeJA and stress, we examined gene expression under aluminum and MeJA exposures (Figures 5D–E). *CsSCPL13-IA* and *14-IA* were markedly induced by Al^3+^ treatment, whereas only low expression levels of *CsSCPL2-IA, 5-IA*, and *11-IA* were detected (Figure 5D). *CsSCPL2-IA* expression increased gradually after 12, 24, and 48 h under MeJA treatment whereas *CsSCPL11-IA, 13-IA*, and *14-IA* increased up to 24 h and decreased thereafter as compared to the control (Figure 5E). On the other hand, *CsSCPL5-IA* expression was remarkably reduced upon exposure to MeJA compared to the control. The *CsSCPL11-IA* expression maximum was observed under MeJA exposure.

### Cloning and Characterization of *CsSCPL-I* Genes

**FIGURE 6:**
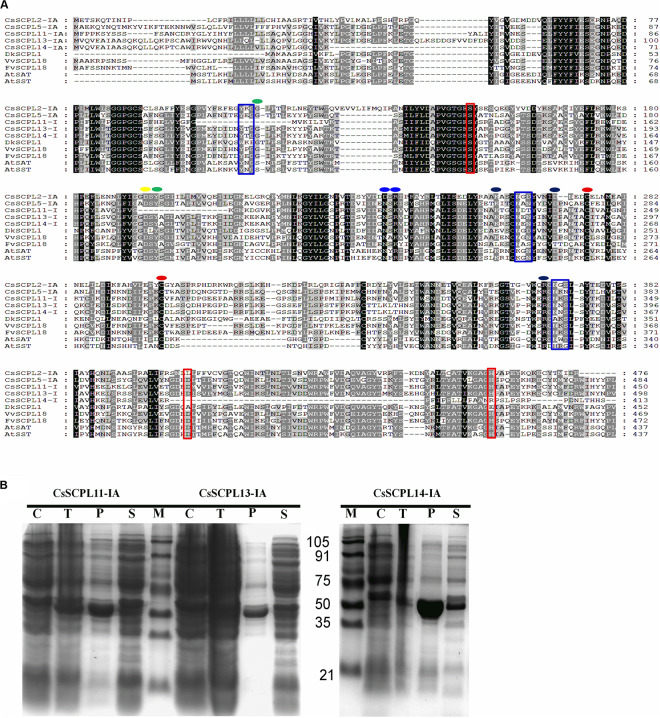
**(A)** Aligment of CsSCPL-I with characterized SCPLs from other species. Conserved amino acid residues participating in key roles are indicated: Cys residues that are likely to form the intersubunit disulphide bond (red circles), oxyanion hole (green circles), recognition of the sinapoyl moiety of the donor molecule (blue circles), the hydrogen bond network (yellow circle), the primary L-malate recognition (black circle), amino acid tracts corresponding to N-glycosylation sites (blue squares), and the amino acid residues forming the catalytic triad of serine carboxypeptidases (S, D, H) (red squares) (cf. Milkowski and Strack, 2004; Chiu et al., 2016). **(B)** The purified His-tagged CsSCPL11-IA (truncated), 13-IA (complete) and 14-IA (truncated) protein fusion recombinants expressed in *E. coli* and partially purified with nickel resin. Proteins were resolved on sodium dodecyl sulfate–polyacrylamide gel electrophoresis (SDS-PAGE), followed by Coomassie Blue R-250 staining.

The five CsSCPL-I genes chosen above for expression analysis were cloned in order to produce recombinant proteins in *E. coli*. However, only three of them (*CsSCPL11-IA, CsSCPL13-IA*, and *CsSCPL14-IA*) expressed successfully in *E. coli*, and therefore chosen for further functional characterization. CsSCPL11-IA, 13-IA and 14-IA proteins contain respectively 450, 498 and 413 amino acid residues, and their calculated molecular weights are 51, 56, and 46 kD (Supplementary Dataset S1). They contain the conserved serine carboxypeptidase motif (Figure 6A) and are generally highly similar to other plant SCPLs. As a typical example in clade IA, the *CsSCPL17-IA* coding region shows > 50% identity with *AtSAT, AtSST, DkSCPL, VvSCPL18*, and *FvSCPL18*. On the other hand, the *CsSCPL14-IA* protein sequence showed < 50% similarity with *AtSAT, AtSST*, and *CsSCPL17-IA* (Figure 6A, Supplementary Dataset S5). Hydropathy analysis revealed that CsSCPL proteins possess a strong hydropathic region near their N-termini, which indicates the presence of a signal peptide.

### CsSCPL-I Proteins Convert Epicatechin (EC) and Epigallocatechin (EGC) to Their Gallate Forms

**FIGURE 7:**
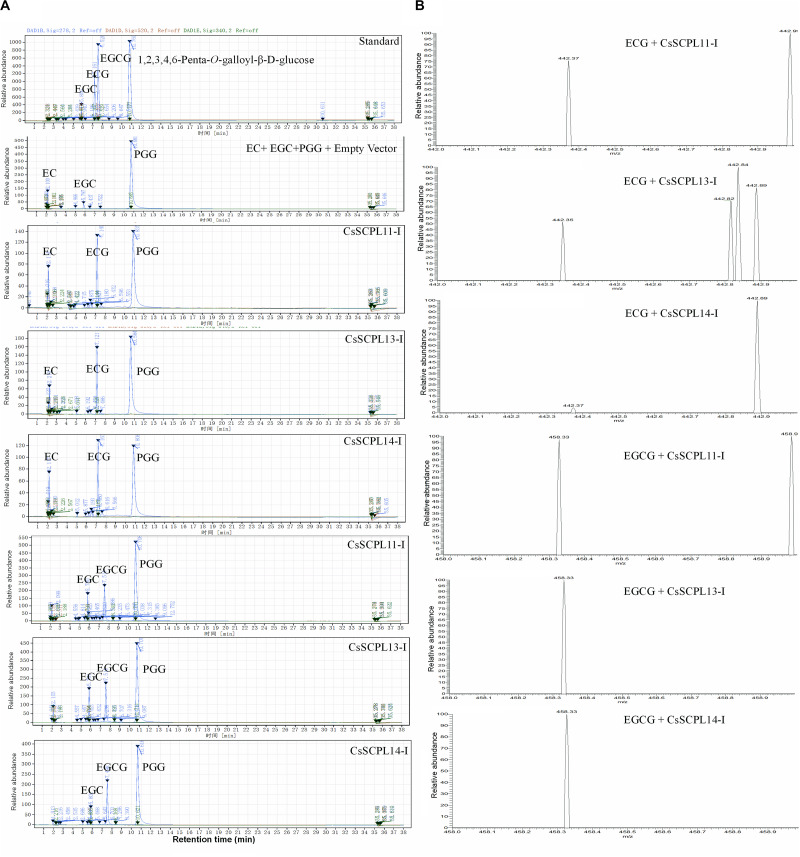
Activity assay of recombinant CsSCPL11-IA, 13-IA, and 14-IA. **(A)** HPLC verification of activity assay with epicatechine (EC), epigallocatechine (EGC) and 1,2,3,4,6-Penta-O-galloyl-β-D-glucose (PGG). **(B)** LC-MS spectra of CsSCPL11-IA, L13-IA and 14-IA activity assay products. ECG, Epicatechin gallate; EGCG, Epigallocatechin gallate. Analytical data were obtained from atleast three biological replicates.

Among the aforementioned five genes selected and expressed in His-tagged fusion proteins in *E. coli* strain BL21 (DE3), only CsSCPL13-IA and the truncated CsSCPL11-IA and 14-IA with their *N*-terminal 50 amino acids deleted were successfully expressed and purified with nickel-resin column. The reason for this deletion is that CsSCPL11-IA and 14-IA contain an *N*-terminal transmembrane domain (Figure 6B, Supplementary Figure S2A). All recombinant proteins successfully expressed showed maximum activity at pH 6.0 with 1,2,3,4,6-pentagalloylglucose (PGG) as an acyl-donor and catechin (C), epicatechin (EC), or gallocatechin (GC) as acceptor substrates. We also used PGG as a donor and C, GC, EC, or EGC as acceptor substrates to assay their activities with partially purified recombinant enzymes. CsSCPL11-IA, 13-IA, and 14-IA preferred PGG as the galloyl donor to convert EC or EGC to ECG or EGCG, respectively (Figure 7, Supplementary Figures S3, S4). High-pressure liquid chromatography (HPLC) coupled with tandem mass spectrometry was used to analyze the reaction and confirm the reaction product with authentic standards (Figure 7A, Supplementary Figures S3, S4).

**FIGURE 8:**
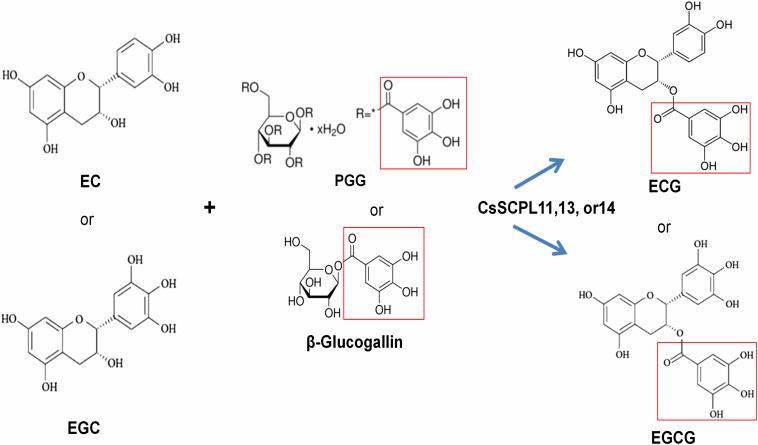
The proposed working model for CsSCPL11,13,14 recombinent proteins-catalyzed galloylation of EC and EGC by using 1,2,3,4,6-penta-O-galloyl-β-D-glucose (PGG) or β-glucogallin.

The kinetic parameters of these three enzymes were calculated through the Lineweaver–Burk plotting method. All of them showed the same trends toward substrate specificity, although the values of kinetic parameters were quite different. CsSCPL13-IA showed higher K_M_ (160.41 μM) and V_Max_ (37.04 μmol mg^–1^ min^–1^) for EC than the other enzymes (Table 1). Meanwhile, CsSCPL14-IA showed maximum K_M_ (103.38 μM) and V_Max_ (62.50 μmol mg^–1^ min^–1^) for EGC (Table 1) at the saturated substrate concentration. The maximum specificity constant (K_cat_/K_M_) was observed for CsSCPL14-IA with EC as the substrate (5.19 s^–1^ μM^–1^) followed by CsSCPL13-IA (3.85 s^–1^ μM^–1^) and CsSCPL11-IA (3.14 s^–1^ μM^–1^), meaning that CsSCPL14-IA has a higher binding affinity for EC. Similar trend was observed for EGC, which showed maximum K_cat_/K_M_ (10.08 s^–1^ μM^–1^) for CsSCPL14-IA, followed by CsSCPL13-IA (6.84 s^–1^ μM^–1^) and CsSCPL11-IA (5.39 s^–1^ μM^–1^). However, the K_M_ of CsSCPL13-IA for PGG was lower than the other two enzymes. The maximum K_M_ (20.81 μM) and V_Max_ (10.64 μmol mg^–1^ min^–1^) values for PGG were observed for CsSCPL14-IA when EGC was used as acceptor (Table 1 and Figure 8). The K_cat_/K_M_ of CsSCPL14-IA was higher for PGG, suggesting that EGC is the preferred substrate for this enzyme.

**TABLE 1:** Acyltransferase kinetics of the recombinant SCPL11-IA, 13-IA, and 14-IA with different substrates.

Substrate	Enzyme	K_M_ (μM)	Vmax (μmol mg^–1^min^–1^)	Kcat (s^–1^)	Kcat/Km (s^–1^ μM^–1^)
EC	*CsSCPL*11−*IA*	85.39 ± 10.62	16.13 ± 2.26	268.82 ± 22.76	3.14 ± 0.64
	*CsSCPL*13−*IA*	160.41 ± 21.2	37.04 ± 5.25	617.28 ± 62.48	3.85 ± 0.42
	*CsSCPL*14−*IA*	64.28 ± 0.81	20.00 ± 4.65	333.33 ± 35.25	5.19 ± 0.48
EGC	*CsSCPL*11−*IA*	40.67 ± 5.56	13.16 ± 1.64	219.30 ± 31.74	5.39 ± 0.81
	*CsSCPL*13−*IA*	49.73 ± 8.36	20.41 ± 3.95	340.14 ± 45.43	6.84 ± 0.94
	*CsSCPL*14−*IA*	103.38 ± 15.63	62.50 ± 5.62	1041.67 ± 146.8	10.08 ± 1.64
PGG	*CsSCPL*11−*IA*	13.34 ± 1.78	4.05 ± 0.62	67.48 ± 7.34	5.06 ± 0.97
	*CsSCPL*13−*IA*	10.43 ± 1.25	3.67 ± 0.52	61.05 ± 8.82	5.85 ± 0.84
	*CsSCPL*14−*IA*	20.81 ± 3.51	10.64 ± 1.44	177.31 ± 22.76	8.52 ± 1.38

*Analytical data were obtained from atleast three biological replicates and are expressed as means ± SD. EGC was used as an acceptor when kinetic parameters were measured for PGG. EC, epicatechin; EGC, epigallocatechin; PGG, 1,2,3,4,6-penta-*O*-galloyl-β-D-glucose.*

## Discussion

A large eudicot family of serine carboxypeptidase-like (SCPL) acyltransferases was originally recruited from a more ancient serine carboxypeptidase family and adapted to take over acyl transfering functions. SCPL acyltransferases have a catalytic triad formed by a nucleophile, an acid and a histidine residue acting as a charge relay system for the nucleophilic attack on amide or ester bonds (Milkowski and Strack, 2004). Although BAHD enzymes have been known for a long time to acylate anthocyanins and flavan-3-ols (Zhao, 2015), the molecular identities of the enzymes catalyzing transacylations from 1-*O*-β-glucose esters or their involvement in the biosynthesis of varied phenolic compounds were not described until the 1990s (Fujiwara et al., 1998; Niemetz and Gross, 2005). Serine carboxypeptidases (S) catalyze the C-terminal peptide bond in proteins and make the Ser-Asp-His catalytic triad. Many studies showed that SCPL proteins share high sequence similarity with the α/β hydrolase (SCP) family, but they did not have the same hydrolase function as SCPs (Milkowski et al., 2004). Instead, SCPLs show acyltransferase and lyase activities (Li and Steffens, 2000; Shirley et al., 2001). For example, the acyltransferase activity of SCPLs catalyzes the formation of the acylsugar 2,3,4-isobutyryl-glucose (Lehfeldt et al., 2000), while sinapate ester is synthesized in *A. thaliana* by the activity of sinapoyl-glucose:malate sinapoyltransferase and sinapoylglucose:choline sinapoyltransferase (Fraser et al., 2005). The present study is the first analysis that provides comprehensive details of SCPLs at the genomic, evolutionary, and catalytic levels in *C. sinensis*.

### Genome-Wide Analysis of SCPL Gene Family in Tea Plant Genome

SCPL acyltransferases play critical roles in many crops, and several of them have been identified and characterized, such as in barley (Baulcombe et al., 1987), rice (Washio and Ishikawa, 1994), pea (Jones et al., 1996), *Arabidopsis* (Li et al., 2001), tomato (Moura et al., 2001), persimmon (Ikegami et al., 2007), and poplar (Zhu et al., 2018). However, so far, not a single study has reported a systematic analysis of SCPLs in *C. sinensis*. Herein, we report a genome-wide identification of SCPL family members, gene expression analysis, and enzymatic assays of select members to explore their substrate specificities and provide clues about their potential functions. We found 47 CsSCPLs distributed into three main phylogenetic classes. Considering genome sizes and ancestral genome duplication events, a ratio of 2:4 SCPL genes is expected between the poplar and *Arabidopsis* genomes (Tang et al., 2008). However, SCPL genes were distributed in these species at the ratio 2:1 in each of the phylogenetic classes (Zhu et al., 2018). This result suggests that SCPL genes were lost in the Arabidopsis genome. Similarly, in our study, the ratios of three SCPL classes in *C. sinensis* and *Arabidopsis* were 1:0.7, 1:1.3, and 1:2.5. The high ratio displayed in *C. sinensis* for the CsSCPL-I class is significant and indicative of genome duplication events. The paralogous pairs, *CsSCPL16-IA/20-IA*, and *12-IA/8-IA* show K_s_ values of 0.21 and 0.36, respectively (Supplementary Table S3), which is similar to the with values of the salicoid lineage duplication event (0.27) that occurred 35 MYA (Guo et al., 2014; Wei et al., 2018). Meanwhile, the other paralogous pairs have lower K_s_ values and might have originated from recent tandem replication events (Wei et al., 2018). We did not find the evidence of CsSCPL-I paralogous pairs deriving from the ancient γ genome triplication event due to the absence of paralogous with K_s_ values close to that expected for this event (Guo et al., 2014).

Our selection pressure analyses using different models revealed that several codon sites in each branch of the SCPL phylogenetic tree were under positive selection. About 5% (26 out of 476 codons) were under positive or purifying selection in CsSCPL-I genes. Moreover, our analysis revealed an interesting evolutionary dynamic in the CsSCPL-I clade. Grounded on K_a_/K_s_ values, many of these genes are under purifying selection, indicating strong selection pressures after the gene duplication event (Supplementary Table S3). This observation showed that the variation in expression patterns of duplicated genes may be evolving toward novel functions after duplication. It is believed that galloylated catechins contents in modern tea plant cultivars are one of domestication traits (Wei et al., 2018). Consistent with high farmer selection pressures on higher catechins contents, primarily galloylated catechins, in modern tea plant cultivars during thousand years of cultivation history, domestications and evolution, SCPL-I, but not SCPL-II genes were also subject to positive selection presures during the evolution (Supplementary Figure S1).

In our phylogenetic analysis, the number of SCPL genes found in the genomes of different plant species varied in each clade. This finding suggests that the different species studied exhibit conserved evolution and that the gene family evolved in multiple directions in these lineages. The gene clustering events observed in two blocks of scaffold2990 suggest the CsSCPL-I clade evolved more rapidly compared to the other two branches. The exon/intron arrangement was similar within the same clade (Du et al., 2012) and the modal length of the first two exons is well conserved, as observed in Vitis and Arabidopsis (Matus et al., 2008). The phylogenetic distribution of genes in our analysis corroborated previous reports. However, a few members showed distinct exon/intron arrangements, indicating that these SCPL genes may play different functions. CsSCPL genes contain no more than 14 exons, which is comparable to the exon numbers in *O. sativa* and *A. thaliana* SCPL genes. Therefore, we conclude that the SCPL family members in *C. sinensis* maintained their exon/intron structures over the course the evolution, unlike what happened in poplar (Zhu et al., 2018).

Interestingly, some *CsSCPL-I* paralogous pairs were dissimilar in their exon/intron structure, indicating divergence at their gene architecture. Moreover, the conserved motifs found in *CsSCPL* genes shared similar characteristics that indicate a close evolutionary relationship in the family, especially within the same class. The same motif structures found in paralogous pairs reveal that CsSCPL proteins are potentially functionally redundant, whereas, on the other hand, differences in motif number imply functional variation and divergence. Genome duplication events can potentially lead to the modification of gene properties and significantly increase functional variation (Liu et al., 2014; Ahmad et al., 2019). Two paralogous pairs showed K_a_/K_s_ ratios significantly < 1, indicating that they are under strong purifying process and contained highly conserved amino acid residues whereas the three remaining paralogous pairs had K_a_/K_s_ ratios significantly > 1, suggesting that tight evolutionary constraints are under effect to sustain their stability.

### Identification and Characterization of Genes Putatively Involved in Catechins Galloylation

Serine carboxypeptidase-like proteins are involved in the biosynthesis of secondary metabolites conferring tolerance to biotic and abiotic stresses (Wilson et al., 2016). Our study clearly shows that most CsSCPL1As share high similarity in enzyme structure, gene expression pattern, regulatory mechanism, and likely enzymatic function. As a result of whole-genome and recent tandem gene duplication events, the expanded CsSCPL1A genes should play key roles in the formation of tea characteristic secondary metabolites, under both environmental and farmer selection pressures for more galloylated catechins contents in shoot tips. While it is difficult to distinguish clear or subtle differences between these CsSCPL genes, that still undergo convergent or divergent evolution under natural or artificial selection, we further combined gene expression and catechins metabolite correlation analyses to deciphor CsSCPL1As for putative functional differences. Both developmental and environmental factors drastically regulate catechins biosyntesis and accumulation, thus, gene expression patterns in tea plant tissues under normal or stressful environments were analyzed concerning catechins accumulation (Figures 4, 5). These data betrayed their generally similar but individually differential roles and putatively diverse functions.

Our sequence profiling revealed conserved regulatory *cis*-acting elements present in CsSCPL-I gene promoters. Their modular compositions varied among the CsSCPL-I members, potentially coordinating responses to complex stimuli, such as light, hormones, and biotic and abiotic stresses. The analysis of transcriptional patterns can offer clues to explore gene functions. Expression of CsSCPL genes was assessed in different tissues and suggested functional differences that *CsSCPL* genes play *in planta*. In addition, about 61% (17/28) of the *CsSCPL* genes showed high expression in young leaves while 57% (16/28) was highly expressed in the apical bud, suggesting they play roles in these organs. Our qRT-PCR analysis of selected CsSCPL genes was consistent with the public RNA-Seq data analyzed. CsSCPL expression generally increased in response to Al^3+^ and MeJA exposures, except for *CsSCPL11-IA*, which expression was repressed in response to MeJA (Figure 4). CsSCPL transcriptional activity was induced in response to heat, but it decreased in response to cold, high salinity, and drought stresses (Chiu et al., 2016). Studies in rice SCPLs suggested that OsBISCPL1 plays a role in defense against multiple stresses (Liu et al., 2008).

It has been speculated that Cs*SCPL-I* genes are involved in the synthesis of galloylated catechins in young leaves and the apical bud for defense against insects or pathogens in the tea plant (Wei et al., 2018). Each member of the paralogous pairs *CsSCPL9-I/11-IA, CsSCPL15-I/10-IA*, and *CsSCPL19-IA/1-IA* shows distinct expression patterns and gene architectures, implying that they may be undergoing functional divergence. On the other hand, *CsSCPL27-IA/24-IA* and *CsSCPL14-IA/24-IA* showed, in addition to sequence similarity, very similar expression patterns and gene structures, strongly indicating functional redundancy. Cs*SCPL2-IA* and CsSCPL*11-IA* were highly induced under MeJA treatment. Therefore, these genes may be interesting candidates for playing roles in disease resistance mechanisms.

The biosynthesis of galloylated catechins is based on galloyl transacylation reactions at the 3-position in the C ring of non-galloylated catechins. The transacylation reactions are accomplished via activated donor molecules in plants. Coenzyme A thioesters in the BAHD family and 1-*O*-glucose esters in SCPLs serve as activated donors due to their high free energy of hydrolysis (Fraser et al., 2007). Among 1-*O*-glucose gallic esters, PGG or its hydrolyzing products, such as di-, tri-, tetra-, and mono-galloylglucoses, such as β-glucogallin, can act as galloyl donors used to modify EC or EGC to form ECG or EGCG, respectively. Tea plant leaves produce many polygalloylated glucose derivatives (Yang and Tomás-Barberán, 2018; Wei et al., 2019). β-Glucogallin is generated from UDP-glucose and gallic acid in tea plants by UDP-glucose: galloyl-1-O-β-D-glucosyltransferase CsUGT84A22 (Liu et al., 2012), whose homologs were also reported in several other plants (Ono et al., 2016; Tahara et al., 2018; Zhao et al., 2020). β-Glucogallin is further used as the galloyl donor in the sequential galloylation of 1,6-di-O-galloyl-β-D-glucose to generate 1,2,6- tri-, 1,2,3,6- tetra-, and 1,2,3,4,6-penta-O-galloyl-β-D-glucose (Niemetz and Gross, 2005). In the biosynthesis of gallotannins, 1,6-di-O-galloyl-glucose: 1,6-di-O-galloylglucose 2-O-galloyltransferase activity was also detected, suggesting that 1,6-di-O-galloyl-glucose can also act as an acyl donor (Niemetz and Gross, 2005).

On the other hand, hydrolyzable tannins, including PGG (pentagalloylglucose) and EGCG, can be readily hydrolyzed enzymatically to generate series of galloylated glucose derivatives, such as di-, tri-, tetra-, and mono-galloylglucoses, as well as gallic acid and catechins, respectively, by many organisms, such as plants and microbes (Jana et al., 2014; Dai et al., 2020; Zhao et al., 2020). Indeed, our assay showed that empty vector control displayed significant hydrolysis of PGG (Figure 7). That explains why EGCG in tea plant leaves is not stable, so did PGG, and perhaps, why PGG can be used as acyl donors. Tea plant leaves accumulated high levels of di-, tri-, tetra-, and mono-galloylglucoses^12^ (Yang and Tomás-Barberán, 2018; Wei et al., 2019).

Microorganisms, such as bacteria and phytopathogens, have particularly active enzyme tannases or hydrolyzable tannin hydrolases, to overcome the plant chemical defense and utilize energy provided by plants (Jana et al., 2014). Therefore, the partially purified enzyme extracts in this study might contain some bacterial tannase-activity proteins, could trigger the hydrolysis of PGG into various galloyl glucoses that may act as proper acyl donors for the catechins galloylations, which could also occur in tea plants. Nevertheless, our work is the first study to display galloylated catechin synthesis through an enzymatic assay catalyzed by CsSCPLs from in tea plants. Previously, two reaction steps of an enzyme from plant extracts was carried out, involving UGGT and another involving ECGT in *C. sinensis* and used β-glucogallin as a donor molecule for the transacylation reactions (Liu et al., 2012). But the gene coding the enzyme is not identified yet.

CsSCPL enzymes are hydrophobic glycoproteins, and more than one serine residue, rather than metal ions, is involved in their active sites. Only a few studies e.g., (Ikegami et al., 2007; Terrier et al., 2009, in persimmon and grapevine, respectively) have investigated at the genetic level SCPL acyltransferases, which are most likely involved in the biosynthesis of galloylated catechins. However, their enzymatic functions have not been verified, and the correlations with galloylation of catechins were only hypothetical. Five candidate genes clustered into the CsSCPL-I clade capable of catalyzing the formation of glucose esters were selected from the *C. sinensis* genome. Their functional analyses were assessed through enzyme activity assays. Among them, three genes were successfully expressed in *E. coli* and showed higher binding affinity to EGC than EC. The discrepancies between the catalytic properties observed in our study and that reported by Liu et al. (2012) may be due to the substrate used for the reactions or the enzyme sources since that study used plant extracts whereas we used purified recombinant enzymes.

### Three SCPL-IA Enzymes Are Involved in the Biosynthesis of Galloylated Catechins

The availability of the tea genome sequence enabled the identification of the IA subclade of the SCPL acyltransferase gene family, which is likely involved in synthesizing major portions of monomeric galloylated catechins in the leaves (Wei et al., 2018). Unlike the insoluble polymerized proanthocyanidins (PAs, or condensed tannins) that are primarily present in the majority of other plant groups, tea leaves mainly accumulate soluble monomeric catechins (e.g., C, GC, EC, EGC) and their galloylation derivatives (CG, ECG, GCG, EGCG) instead (Wei et al., 2015). These molecules account for up to 75% of the total catechins in leaves and have a major impact on tea quality. However, the genetic basis for the biosynthesis of galloylated catechins is not fully understood, which currently one of the fundamental biological questions in tea biology (Zhao et al., 2020). Biochemical studies demonstrated that the biosynthesis of flavan-3-ols via 1-*O*-glucose ester-dependent reactions are catalyzed by galloyl-1-*O*-β-D-glucosyltransferase (UGGT) and that epicatechin:1-*O*-galloyl-β-D-glucose *O*-galloyltransferase (ECGT) (Liu et al., 2012). Similar reactions have been found in other plant species, such as grapevine and persimmon (Ikegami et al., 2007; Terrier et al., 2009; Wilson et al., 2016). So far, among at least 22 CsSCPL-I genes redundantly present in the tea genome, which have been regarded as the most likely candidates responsible for the galloylation of catechins, no molecular evidence of any gene involved in galloylated catechins biosyntesis in tea plants has been published so far (Wei et al., 2018). Our comprehensive analyses imply that these CsSCPL-I may have overlapping but differential functions. Due to the presence of diverse and complex hydrolyzable tannins, including various *O*-glucose gallic esters and galloylated catechins, CsSCPL-I acyltransferases may have similar but differential enzymatic functions.

We identified and characterized three SCPL-IA genes which expression levels were highly correlated with the accumulation of EGCG and ECG, given their high transcriptional activities in apical buds and young leaves, where most galloylated flavan-3-ols accumulate (Figure 5B). We further confirmed enzymatically that the recombinant enzymes were able to catalyze the production of galloylated catechins *in vitro* (Table 1 and Figure 8). A recent study showed that the galloylated catechins, such as EGCGs, are primarily localized to the central vacuole for storage (Xu et al., 2016). Consistently, our analysis showed that CsSCPL11 and CsSCPL13 are predicted to be localized to the lososomes, including vacuoles (Supplementary Dataset S1). The measured enzymatic activities of these SCPLs were relatively low *in vitro*, considering the high levels of galloylated catechins that accumulate as major forms in tea leaves. Several reasons could explain this result. Firstly, PGG, rather than β-glucogallin, was used as an acyl donor (Figure 8). Although it was shown that PGG could be efficiently hydrolyzed into different galloylglucoses, including β-glucogallin, the enzyme efficiency was low. Secondly, protein modifications (e.g., glycosylation, phosphorylation), or molecular interactions (e.g., homo- or heteromerization, allosteric control) may be required *in celula* to enhance enzyme activity. This is the first report on CsSCPL-I genes responsible for galloylated catechins biosyntesis. Notwithstanding, our results prove the hypothesis that SCPLs are involved in the galloylation of EC or EGC, given that all the three enzymes studied preferred to modify EC or EGC into their galloylated forms. Further studies on these aspects are essential to further our understanding of the roles SCPL enzymes play in plant physiology.

## Conclusion

We demonstrated that both convergent and divergent evolution of CsSCPL1A genes in tea plant genome and their generally similar but differential gene expression patterns in various tea plant tissues as effects of developmental and environmental factors, and that CsSCPL11-IA, 13-IA and 14-IA are SCPL acyltransferases that share similar enzymatic kinetics in the galloylation of EC or EGC. Our genome-wide analysis of the SCPL gene family in tea, and biochemical characterization of three recombinant CsSCPLs, such as substrate specificity and enzymatic kinetic parameters, revealed important results to understand the physiological roles these compounds play in tea. The three CsSCPL genes functionally characterized in the study display distinct expression patterns in different tissues of the plant and response to abiotic stress and hormones. The insights provided by this study will not only help to understand the biosynthesis timing and location of these galloylated metabolites but also which physiological roles that ECG or EGCG play in tea plants.

## Data Availability Statement

Publicly available datasets were analyzed in this study. This data can be found here: The Tea Plant Information Archive – TPIA public database: http://teaplant.org/; http://tpia.teaplant.org/index.html. CsSCPL2-I (TEA009664, NCBI Genbank accession MK843824), CsSCPL5-I (TEA034028; MK843825), CsSCPL11-I (TEA023451; MK843826), CsSCPL13-I (TEA034055; MK843827), and CsSCPL14-I (TEA027270; MK843828)].

## Author Contributions

JZ planned and designed the research. MA, PL, GS, and EX performed experiments and analyzed data. MA, JZ, VB, and XW wrote and edited the manuscript. All authors contributed to the article and approved the submitted version.

## Conflict of Interest

The authors declare that the research was conducted in the absence of any commercial or financial relationships that could be construed as a potential conflict of interest.

## Footnotes

1 http://teaplant.org/

2 https://web.expasy.org/protparam/

3 http://visualization.ritchielab.psu.edu/phenograms/plot

4 http://gsds.cbi.pku.edu.cn

5 http://services.cbu.uib.no/tools/kaks

6 http://classic.datamonkey.org/

7 http://meme-suite.org/

8 http://pfam.xfam.org/

9 http://bioinformatics.psb.ugent.be/webtools/plantcare/html/

10 http://tpia.teaplant.org/

11 https://sourceforge.net/projects/mev-tm4/

12 http://pcsb.ahau.edu.cn:8080/TCDB/f

## Supplementary Material

FIGURE S1 Selection pressures among CsSCPLI and II gene sequences using mechanistic empirical combination (MEC) model.

FIGURE S2 Topological analysis of CsSCPLs and designed expression of truncated proteins.

FIGURE S3 HPLC analyses of CsSCPLs-enzymatic reactions.

FIGURE S4 LC-MS/MS analysis of CsSCPLs-enzymatic products in negative mode.

TABLE S1 Composition of tea plant standard culture solution of Shigeki Konishi.

TABLE S2 Conserved domain analysis of CsSCPL proteins.

TABLE S3 Synteny analysis of CsSCPL gene paralogues.

TABLE S4 Log-likelihood values and statistics for PAML site models of positively selection.

TABLE S5 Positively selected sites under different PAML site models using Bayes empirical analysis.

DATASET S1 Physical parameters of tea proteins coded by CsSCPL genes.

DATASET S2 *Cis*-acting analysis of CsSCPL gene promoter regions.

DATASET S3 Expression level of CsSCPLs in different Tea tissues, response to cold, NaCl, Al3^+^/H^+^ stresses, Shedding, MejA and PEG treatment.

DATASET S4 List of primers of CsSCPL genes used for qRT-PCR validation.

DATASET S5 Identity and similarities at amino-acid level among tea and different characterized SCPLs.
