# Supplementary material for: Genome-Wide Analysis of Serine Carboxypeptidase-Like Acyltransferase Gene Family for Evolution and Characterization of Enzymes Involved in the Biosynthesis of Galloylated Catechins in the Tea Plant (Camellia sinensis)
Source: Front Plant Sci. 2020 Jun 25;11:848. doi: 10.3389/fpls.2020.00848 (PMC7330524; doi:10.3389/fpls.2020.00848)

Supplemental Figure S1. Selection pressures among CsSCPLI and II gene sequences using mechanistic empirical combination (MEC) model.

A. Strong positive selection pressures among CsSCPL I genes

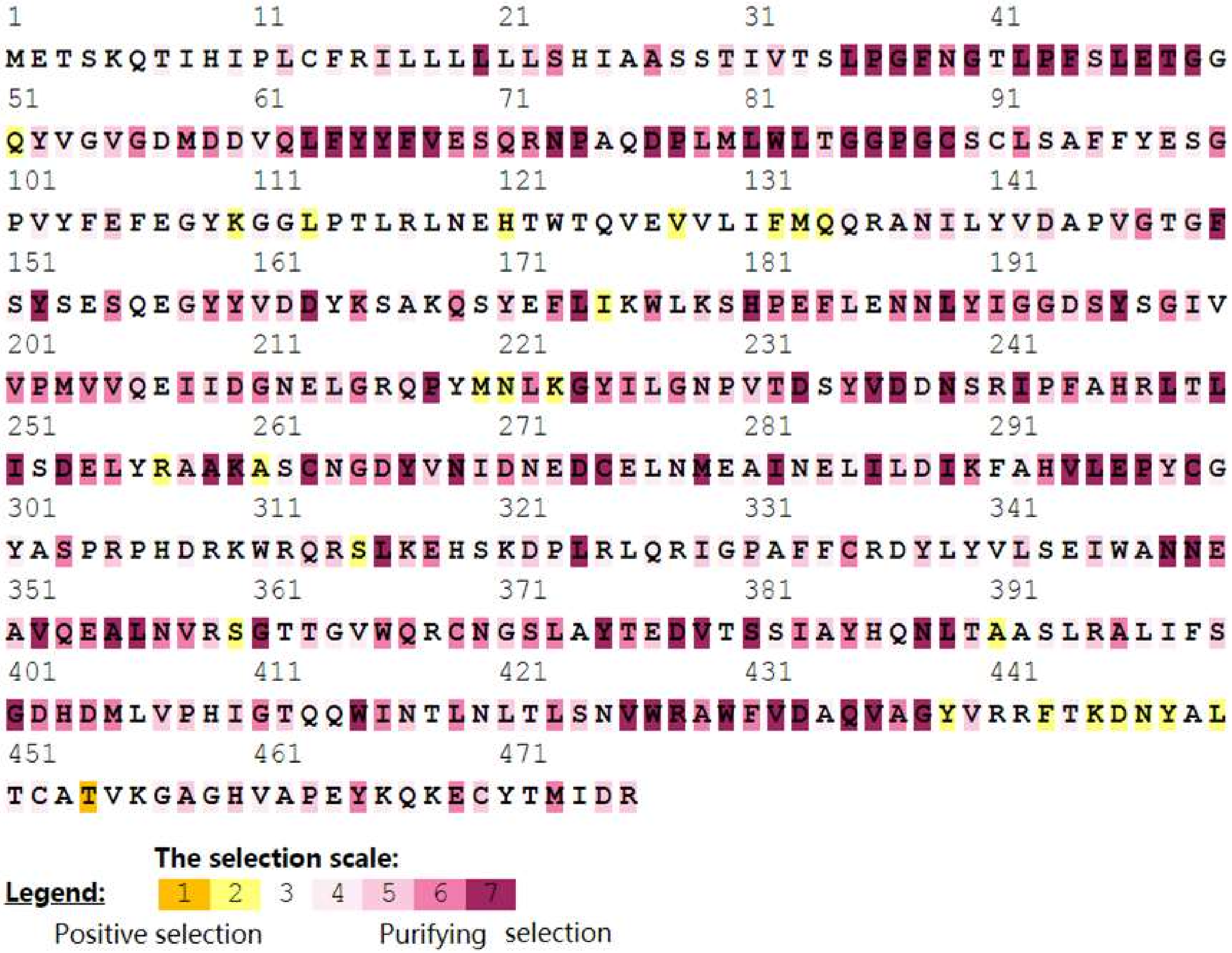

Selection pressures among CsSCPL I gene sequences using mechanistic empirical combination (MEC) model of selection online tool. Brown and Yellow highlights represent positive selection, light purple and white highlights represent neutral selection, and purple highlight represents purifying or negative selection on codons

B. No obvious selection pressures among CsSCPL II gene sequences

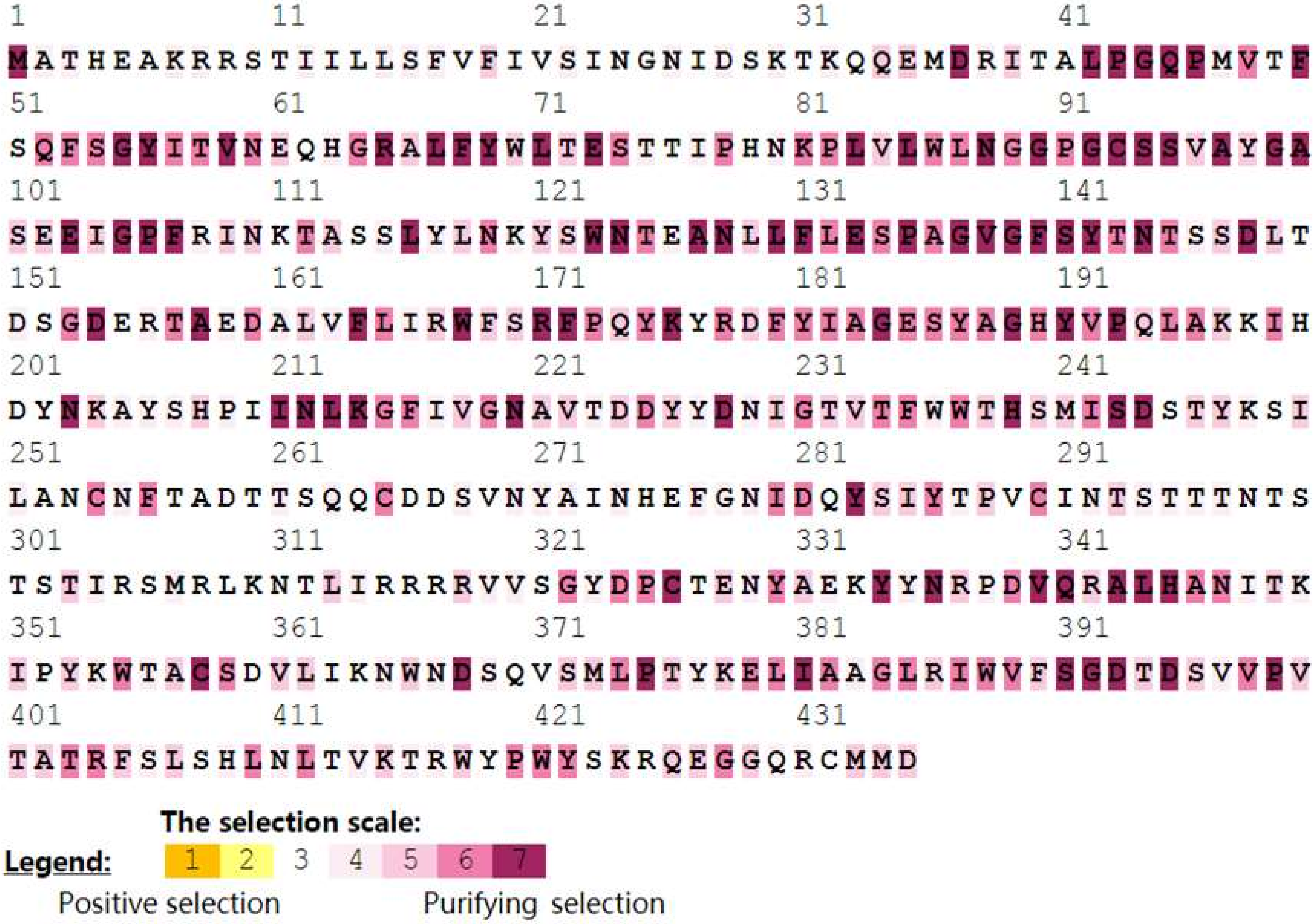

Brown and Yellow highlights represent positive selection, light purple and white highlights represent neutral selection, and purple highlight represents purifying or negative selection on codons

Supplemental Figure S2. Topological analysis of CsSCPLs and designed expression of truncated proteins.

A. Topological analysis of CsSCPLs and designed expression of truncated proteins. The transmembrane domains (TMs) underlined were predicted with the TMHMM Server ver. 2.0 (http://www.cbs.dtu.dk/ services/TMHMM/).

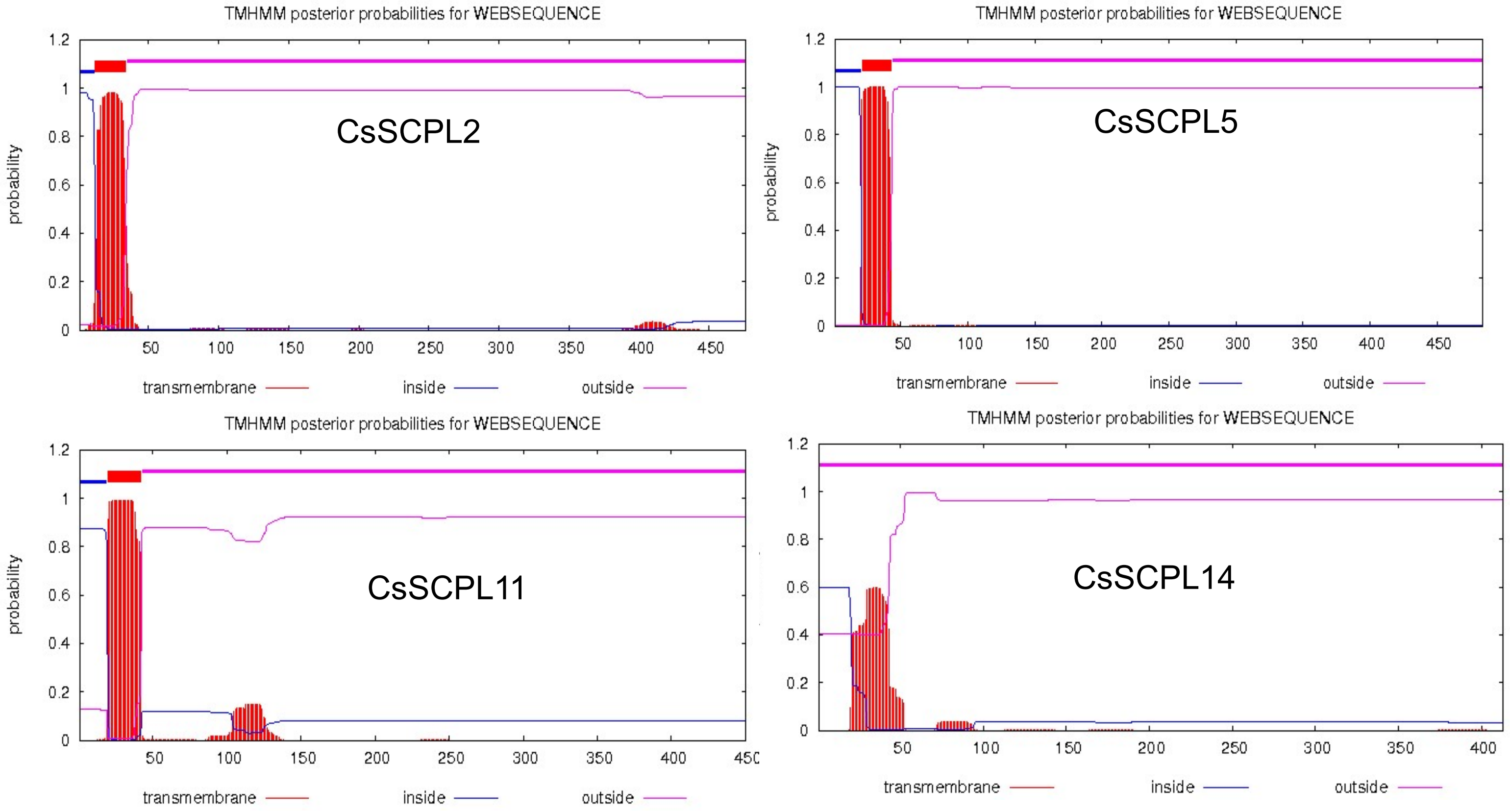

B. Red amino acids showing putative transmembrane regions were deleted in bacterially expressed CsSCPL11 and CsSCPL14.

>TEA023451-SCPL11
MFPPKSYSSSFSANCDRYGLYIHYFLLLLLLSAQAVLGGHIVKYLPGYDG
MELPFKLETGYALIYIRVNESELFYYFIESQGNPQEDPIFLWLPGGPGCSSFHELVYGFGMVKILIVKTASIIFLDAPVGTGFSYSRTADGWPTSDSKSAEQSYQFL
REWFDEHPQYLAVQLFVGKGNKDGVKPFMNLKGYLLGSPRTDSVIDENSKVVFAHRMALISDEIYENAKTGCNDTYVSIDPANTACIVALGNIKTCIKDLFRNDILE
PKCVFATPDPGEEPAARRSLEEGPSDFLLSPPMIPNLWCRNFNYVLSYIWSNDDTVQEVLHVRKGSVLNWERCNKSLSYTKDILTVVPVHEELKELGLEVLVETG
DRDMVVPFVGTVKWIKSLNLTVVNDWRPWFVDGQVAGYTIKYSEHGYRLTYATVKGAGHTAPEYYRMECYYMFDRWIHYYPI
>TEA034055-SCPL13
MVQVEAIAASQKQLLQKPSCAWIRWVQNHLLLQLLLAQPVLGGQIVKYLPGFDGELPFKLETGQLKSDDGFVVDFDRYISVDDSELFYYFIESEGNPQEDPLFLW
LTGGPGCTSFSGLLYEVGPMEYDIDNYTGGLPKLKYYPYARTKTASMIFLDAPVGTGFSYARTPGGWPTSDTKSAEQSYQFLRKWLVEHPQFLSVQLFVGGDSY
AGLAVPLITKKIIDGNKEKAEPYMNIKGYLVGCPGTDSVIDGNSRVDFAHRMALISDEIYENAKRSCNENYINVDPANTACITAMGAVQKCLEDLSDKDILKPKCDLS
SQDHPEGPDRRFLKEGSSEFLLSPSTFPKSWCTTLKLTHNSIWANDDGVQEALHVRKGTVPRWERCNNSLSYTKDVSSVIAVHKELSRSSLEVLVESGDHDISV
PYMGTLKWIKSLNLTLVDDWRPWFVDNQVAGYTMKYSEEHGYHLTFATIKGAGHPAPEYYRRECYCLFDRWINYNPV
>TEA027270-SCPL14
MVQVEAIAASQKQLLQKPTCAWIRWVQNHLLLLLVLLAQPVLGGQIVKYI
MSVDDSSELFYYFIESEGNPQEDPLFLWLTGGPGCTSFSGLLYEVGPMEYDIDNYTGGLPKLKYYPYARTKTASMIFLDAPVGTGFSYARTPGGWPTSDTKSAE
QSYQFLRKWLVEHPQFLSVQLFVGGDSYAGLTVPLITKKIIDGNKEKAKPYMNIKGYLVGCPGTDSVIDGNSRVDFAHRMALISDEIYENAKRSCNENYINVDPANT
ACITAMGAIQKCLKDLSDKDILKPKCDLSSQDHPEGPDRRFLKEGSSEFLLSPSTFPKPWCTTLKLTHNSIWANDDGVQEALHVRKGTVPRWERCNNSLSYTQD
VLSVIAVHKELSRSSLEVLVERFFIFFLLLPAANRPSLPSRPPDNLTHHS

Supplemental Figure S3. HPLC analyses of CsSCPLs-enzymatic reactions

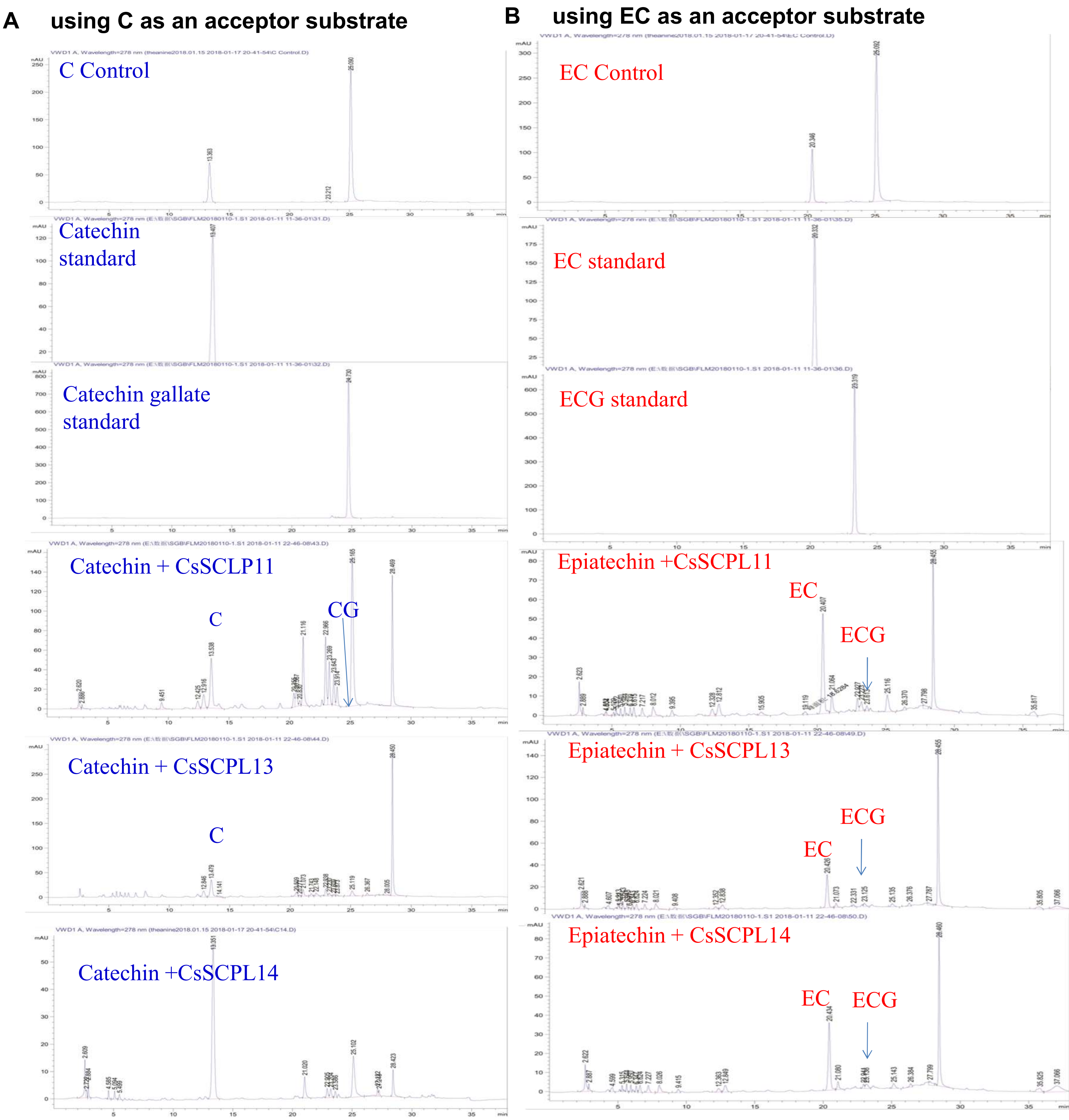

C using GC as an acceptor substrate

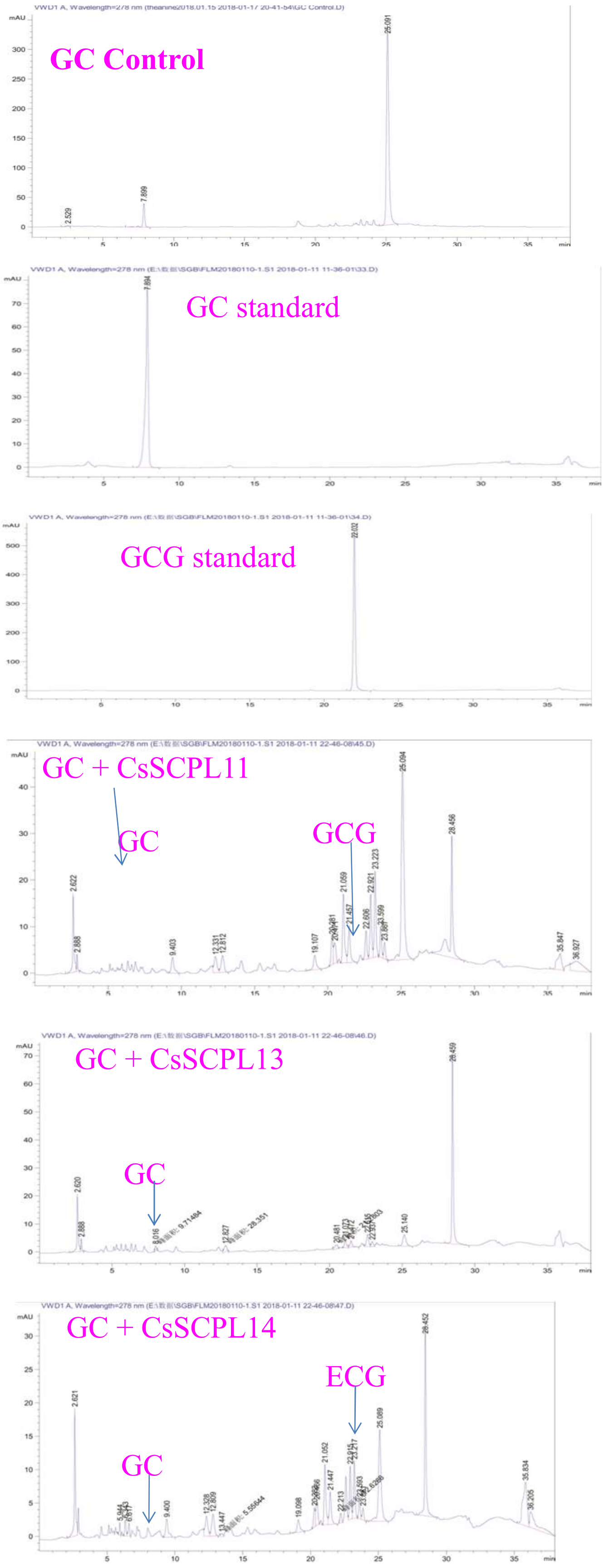

D using EGC as an acceptor substrate

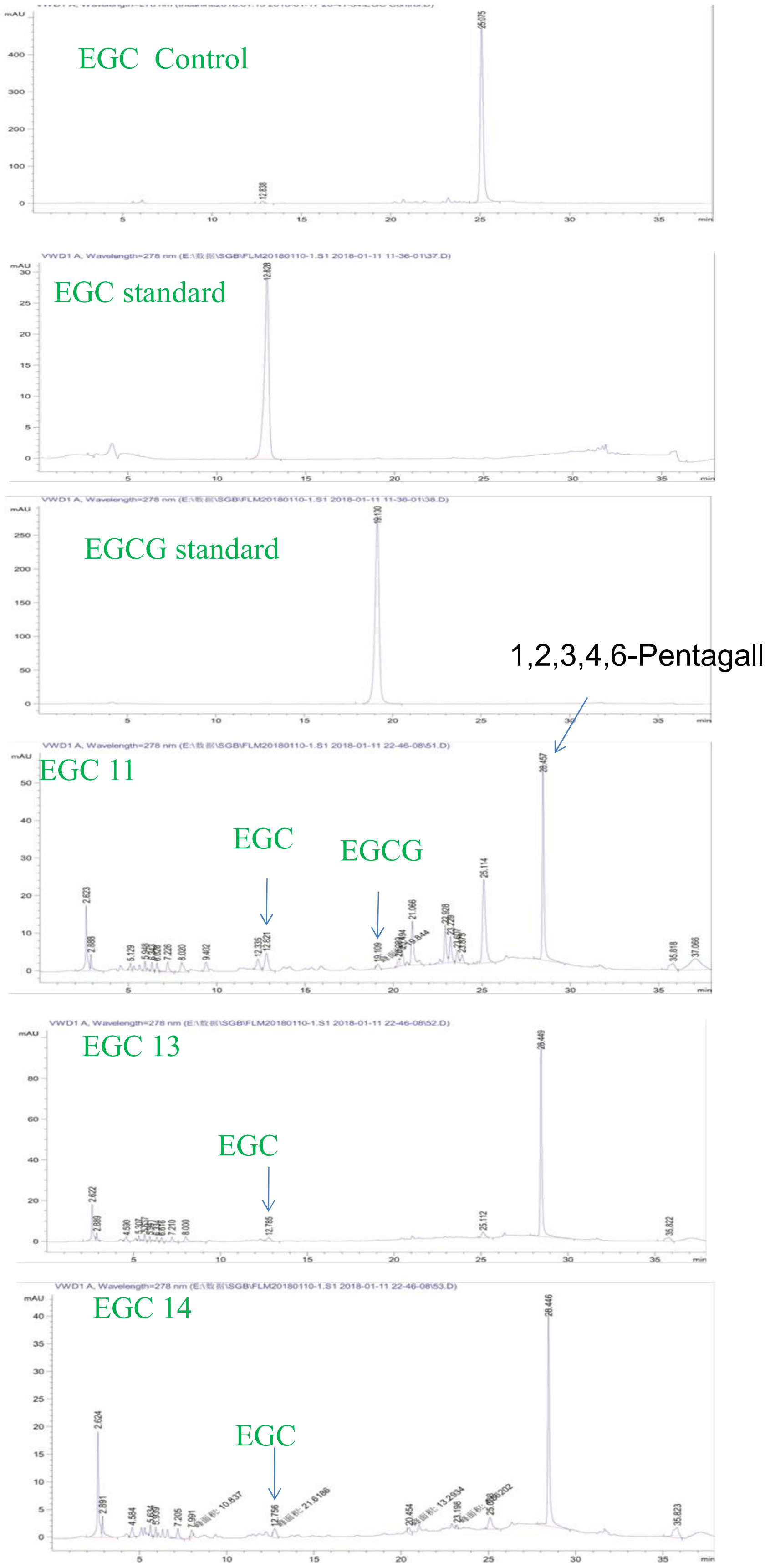

Supplemental Figure S4. LC-MS/MS analysis of CsSCPLs-enzymatic products in in negative mode

A. using C as an acceptor substrate

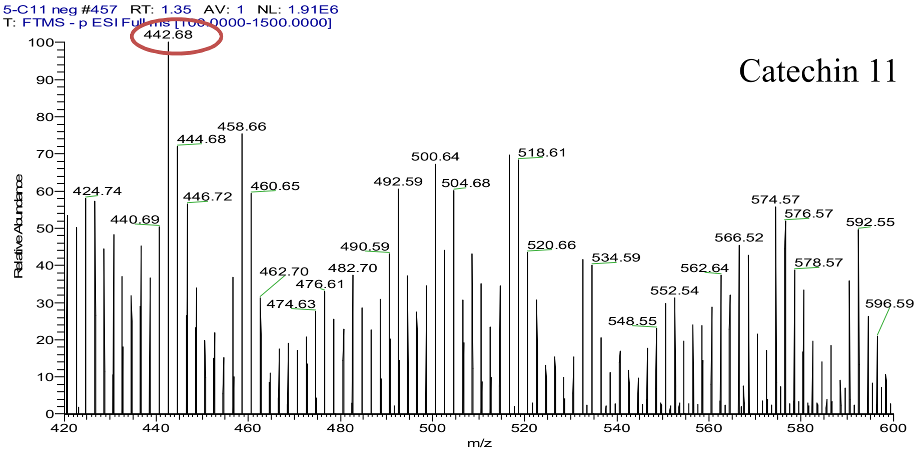

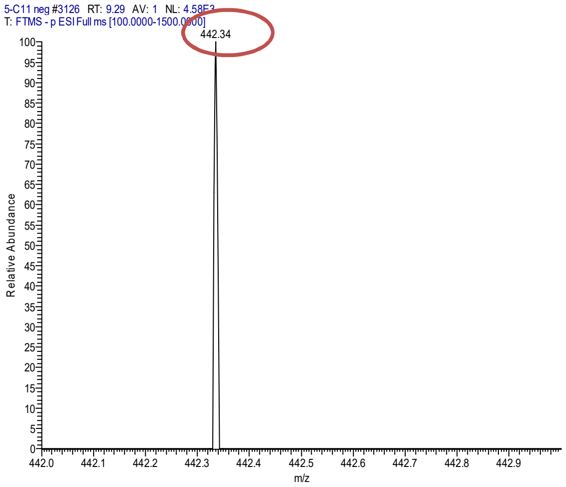

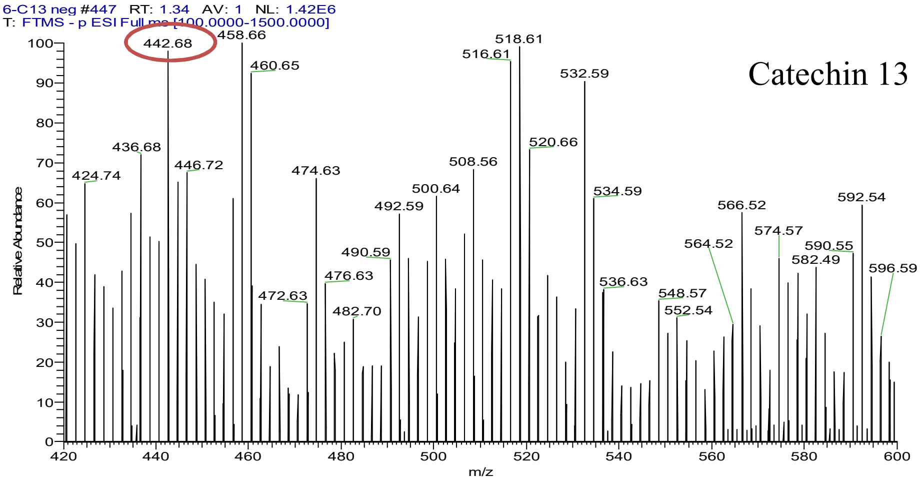

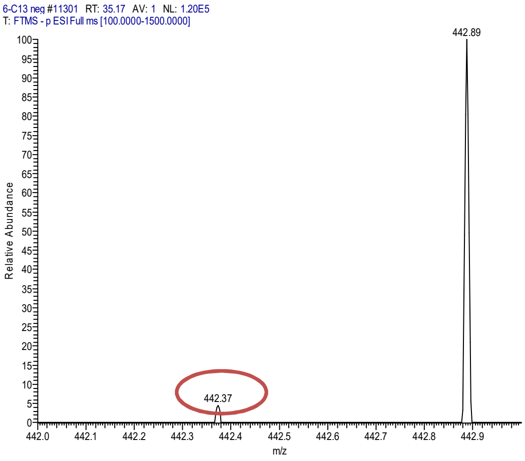

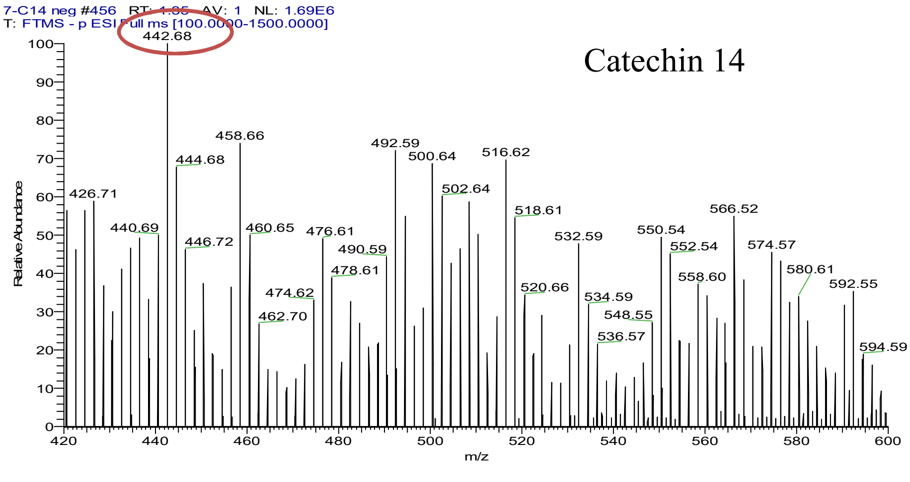

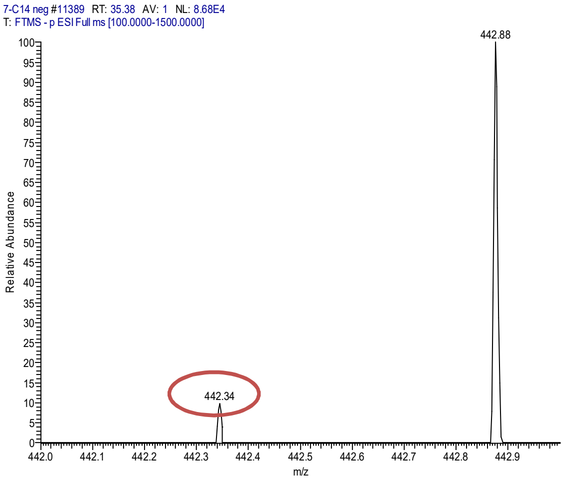

B. using EC as an acceptor substrate

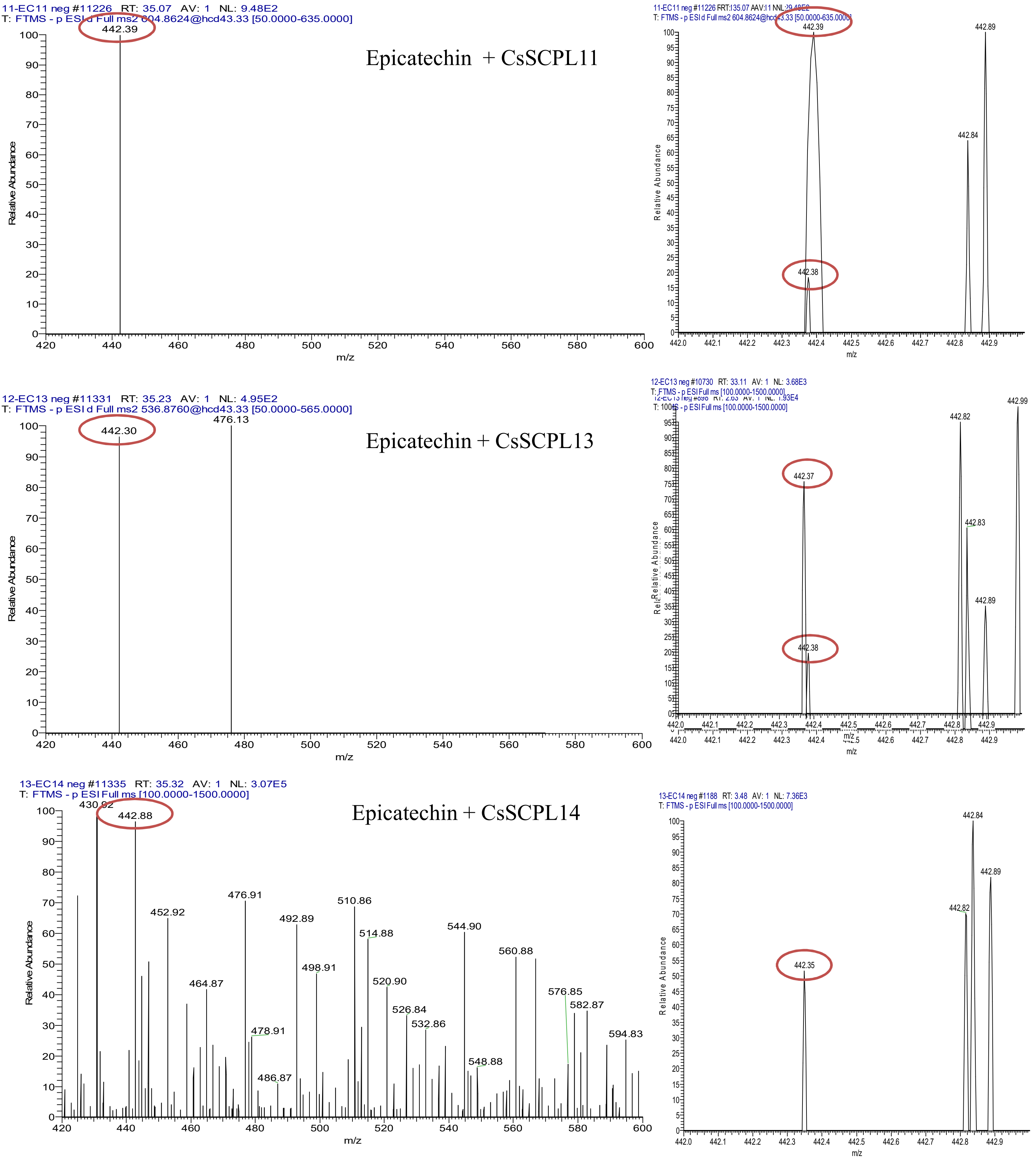

C. using EGC as an acceptor substrate

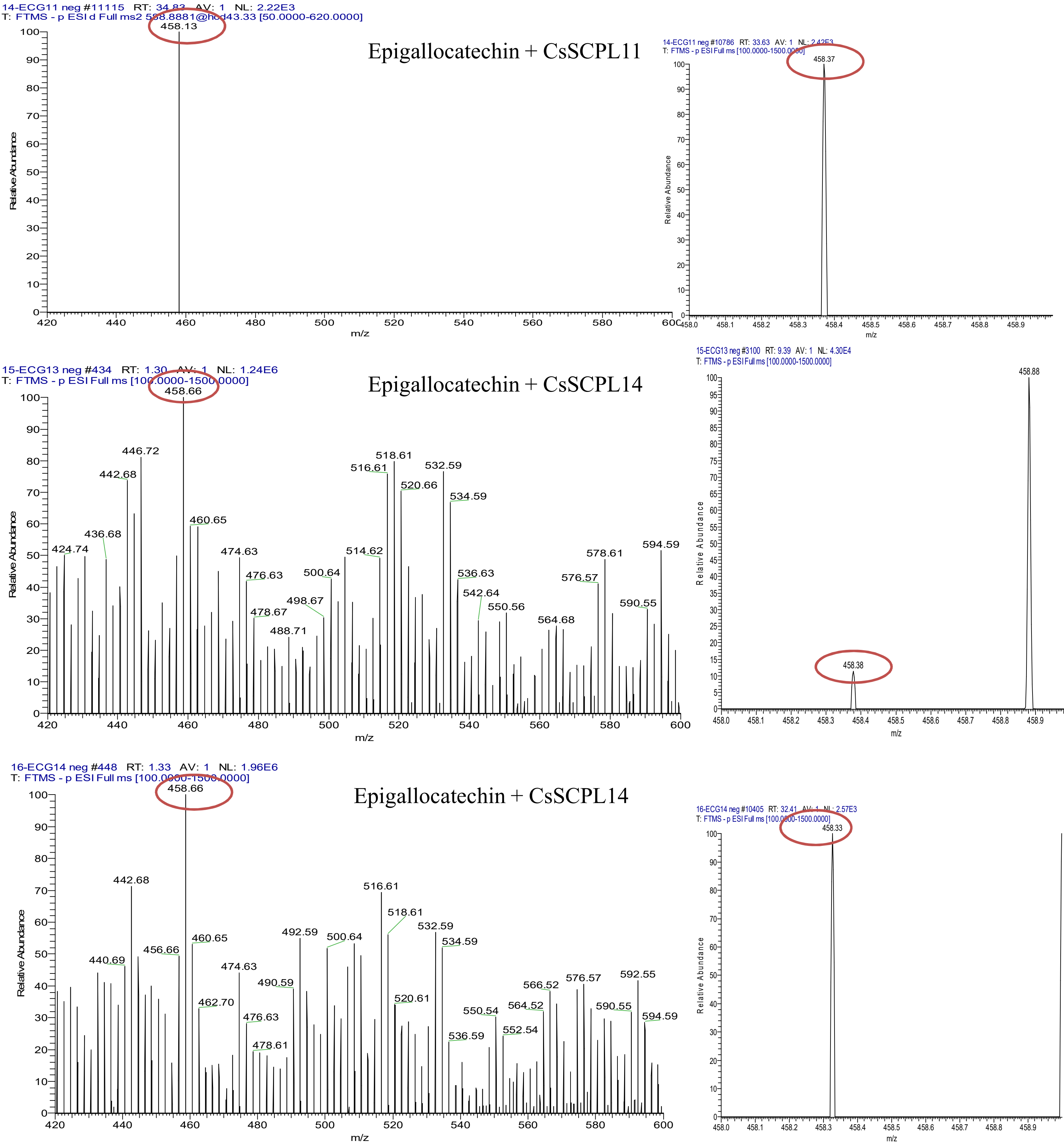

Supplement: FIGURE S1 — Selection pressures among CsSCPLI and II gene sequences using mechanistic empirical combination (MEC) model. [file Presentation_1.pdf]
