# Supplementary material for: Genome-Wide Analysis of Serine Carboxypeptidase-Like Acyltransferase Gene Family for Evolution and Characterization of Enzymes Involved in the Biosynthesis of Galloylated Catechins in the Tea Plant (Camellia sinensis)
Source: Front Plant Sci. 2020 Jun 25;11:848. doi: 10.3389/fpls.2020.00848 (PMC7330524; doi:10.3389/fpls.2020.00848)
Supplement: TABLE S1 — Composition of tea plant standard culture solution of Shigeki Konishi. [file Data_Sheet_2.doc]

**Supplemental Table S1.** Composition of tea plant standard culture solution of Shigeki Konishi

| Element | Compound | | Comcentration(mg/L) |
| --- | --- | --- | --- |
| N | | NH4-(NH4)2SO4 | 30 |
|  | | NO3-NCa(NO3)24H2O | 10 |
| P | | KH2PO4 | 3.1 |
| K | | KH2PO4、K2SO4 | 40 |
| Ca | | CaCl2·2H2O | 20 |
| Mg | | MgSO4·7H2O | 25 |
| Fe | | Fe-EDTA | 0.35 |
| B | | H3BO3 | 0.1 |
| Mn | | MnSO4·4H2O | 1 |
| Zn | | ZnSO4·7H2O | 0.1 |
| Cu | | CuSO4·5H2O | 0.025 |
| Mo | | Na2MoO42H2O | 0.05 |
| AL | | Al2(SO4)3·16-18H2O | 10 |

**Reference:** Konishi S, Miyamoto S, Taki T. (1985). Stimulatory effects of aluminum on tea plants grown under low and high phosphorus supply. Soil Science and Plant Nutrition 31:361-368

**Supplementary Table S2.** Domain analysis of CsSCPL proteins

| **Number** | **Sequence** | **Width** | **Annotation** |
| --- | --- | --- | --- |
| 1 | EHPQYLKNQLFIGGDSYAGITVPLVVQKI | 29 | Serine carboxypeptidase |
| 2 | DPLLLWLNGGPGCSS | 15 | Serine carboxypeptidase |
| 3 | KTANJJFLDAPVGTGFSYSNT | 21 | Serine carboxypeptidase |
| 4 | VYSGDHDMVVPVTGTRYWIKSL | 22 | Serine carboxypeptidase |
| 5 | HEEEADRRSLEEGPSBFLLSPPKIPELWC | 29 | None |
| 6 | GSQLFYYFIESZGNP | 15 | None |
| 7 | IWANDEDVQEALHVR | 15 | None |
| 8 | VNDWRPWFVDGQVGG | 15 | None |
| 9 | GAGHTAPEYKRRECY | 15 | None |
| 10 | GCPRTDSVIBENSKIPFAHRM | 21 | None |

**Supplementary Table S3. Synetic analysis of CsSCPL gene paralogs**

| **No.** | **Paralogous** | **Ka** | **Ks** | **Ka/Ks** | **Duplication** |
| --- | --- | --- | --- | --- | --- |
| 1 | TEA016469(CsSCPL16-IA)-TEA034017(CsSCPL20-IA) | 0.241 | 0.205 | 1.18 | SD |
| 2 | TEA034055(CsSCPL13-IA)-TEA034056(CsSCPL24-IA) | 0.021 | 0.013 | 1.67 | SD |
| 3 | TEA034036(CsSCPL9-IA)-TEA034033(CsSCPL21-IA) | 0.037 | 0.028 | 1.31 | TD |
| 4 | TEA034031(CsSCPL22-IA)-TEA034039(CsSCPL10-IA) | 0.113 | 0.082 | 1.39 | SD |
| 5 | TEA034050(CsSCPL6-IA)-TEA027270(CsSCPL14-IA) | 0.049 | 0.055 | 0.89 | SD |
| 6 | TEA034049(CsSCPL19-IA)-TEA020540(CsSCPL1-IA) | 0.020 | 0.022 | 0.88 | SD |
| 7 | TEA023444(CsSCPL12-IA)-TEA034026(CsSCPL8-IA) | 0.318 | 0.360 | 0.88 | TD |

**Supplementary Table S4.** Log likelihood values and statistics for PAML site models of positively selection

| **Gene** | **No. of residues** | **No. of Seq** | **Model** | **Parameter estimates** | **lnL M7** | **lnL M8** | **2∆lnL** | **PAML** | **FEL** | **IFEL** | **REL** | **SLAC** | **% of sites** |
| --- | --- | --- | --- | --- | --- | --- | --- | --- | --- | --- | --- | --- | --- |
| CsSCPL- I | 1377 | 28 | M7 | p = 1.08  q = 1.02 | -34570.18 | -34527.51 | 85.34^**^ | **92, 238**, 239, *404, 478, 530*, *888, 889*, 921, *922,* 960, *962*, **964, 965**, *966*, 967, 968, 969, **973** | 69, 103, 556, 747, 987, 1002 | 5, 26, 69, 70, 103, 477, 556, **973**, 987, 1002 | 69, 70, 103, **238**, 239, 404, 481, 530, 652, 746, *888, 889*, 921, **973** | 69, 103, 160, 556, 679, 934, 1002 | 6.9 |
|  |  |  | M8 | P0 = 0.93  p = 1.14  q = 1.15  P1= 0.07  **ω1 = 6.12** |  |  |  |  |  |  |  |  |  |
| CsSCPL- II | 1350 | 17 | M7 | p =0.69  q = 2.03 | -19848.863733 | -19848.863907 | 0.0004 | 33, 60, 413 | 10, 13, 25, 33, 100, 380, 564 | 10, 23, 25, 100, 134, 240, 350, 380, 410, ,432, 559 | 144 | 33, 380, 491, 534 | 0.001 |
|  |  |  | M8 | P0 = 0.99  p= 0.69  q= 2.03  P1= 0.00001)  **ω1= 1.0** |  |  |  |  |  |  |  |  |  |

The proportion of sites under positive selection (p1), or under selective constraint (p0), and parameters p and q for the beta distribution. Parameters indicating positive selection are in bold. p: significant at 5% level; p: significant at 1% level. Sites potentially under positive selection identified under model M8 are listed according to the sequence numbering. Positively selected sites with posterior probability >0.9 are italicized, 0.8–0.9 in bold, and < 0.8 in plain text. The test statistic 2Δl is compared to a χ2 distribution with 2 df, critical values 5.99, 9.21, and 13.82 at 5%, 1%, and 0.1% significance, respectively.**Significant at 1% level; *Significant at 5% level.

**Supplementary Table S5.** Positively selected sites under different PAML site models using bayes empirical byes analysis

| **Gene** | **Model** | **Codon** | **Amino acid** | **Posterior probability (ω>1)** | **Post mean ± SE for ω** |
| --- | --- | --- | --- | --- | --- |
| CsSCPL- I | M8: selection, beta+ω | 92 | I | 0.852 | 7.160 ± 3.087 |
|  |  | 238 | L | 0.863 | 7.143 ± 2.962 |
|  |  | 239 | W | 0.737 | 6.071 ± 3.416 |
|  |  | 404 | E | 0.952 | 7.861 ± 2.282 |
|  |  | 478 | K | 0.926 | 7.656 ± 2.527 |
|  |  | 530 | A | 0.998 | 8.166 ± 1.726 |
|  |  | 888 | R | 0.986 | 8.087 ± 1.888 |
|  |  | 889 | R | 0.998 | 8.169 ± 1.726 |
|  |  | 921 | M | 0.531 | 4.729 ± 3.870 |
|  |  | 922 | S | 0.980 | 8.046 ± 1.964 |
|  |  | 960 | K | 0.707 | 6.040 ± 3.654 |
|  |  | 962 | L | 0.964 | 7.933 ± 2.154 |
|  |  | 964 | S | 0.882 | 7.320 ± 2.859 |
|  |  | 965 | G | 0.810 | 6.784 ± 3.263 |
|  |  | 966 | H | 0.932 | 7.701 ± 2.474 |
|  |  | 967 | F | 0.707 | 6.073 ± 3.684 |
|  |  | 968 | I | 0.708 | 5.998 ± 3.625 |
|  |  | 969 | K | 0.696 | 5.973 ± 3.698 |
|  |  | 973 | R | 0.894 | 7.413 ± 2.761 |
| CsSCPL- II | M8: selection, beta+ω |  |  |  |  |
|  |  | 33 | A | 0.575 | 1.177 ± 0.412 |
|  |  | 60 | T | 0.516 | 1.117 ± 0.431 |
|  |  | 413 | S | 0.612 | 1.203 ± 0.420 |
